# Enhancement strategy for effective vascular regeneration following myocardial infarction through a dual stem cell approach

**DOI:** 10.1038/s12276-022-00827-8

**Published:** 2022-08-16

**Authors:** Hyeok Kim, Soon-Jung Park, Jae-Hyun Park, Sunghun Lee, Bong-Woo Park, Soon Min Lee, Ji-Won Hwang, Jin-Ju Kim, Byeongmin Kang, Woo-Sup Sim, Hyo-Jin Kim, Seung Hwan Jeon, Dong-Bin Kim, Jinah Jang, Dong-Woo Cho, Sung-Hwan Moon, Hun-Jun Park, Kiwon Ban

**Affiliations:** 1 Department of Biomedicine & Health Sciences, College of Medicine, The Catholic University of Korea, Seoul, South Korea; 2 Division of Cardiology, Department of Internal Medicine, Seoul St. Mary’s Hospital, The Catholic University of Korea, Seoul, South Korea; 3 Research Institute, T&R Biofab Co., Ltd., Siheung, South Korea; 4 Department of Biomedical Sciences, City University of Hong Kong, Kowloon Tong, Hong Kong; 5 SL BIGEN, Inc., Seongnam, South Korea; 6 Department of Creative IT Engineering and School of Interdisciplinary Bioscience and Bioengineering, Pohang University of Science and Technology, Pohang, South Korea; 7 Department of Urology, College of Medicine, The Catholic University of Korea, Seoul, South Korea; 8 Division of Cardiology, Department of Internal Medicine, The Catholic University of Korea, Seoul, South Korea; 9 Department of Mechanical Engineering, Pohang University of Science and Technology, Pohang, South Korea; 10 Department of Animal Biotechnology, Sangji University, Wonju, South Korea; 11 Cell Death Disease Research Center, College of Medicine, The Catholic University of Korea, Seoul, South Korea

**Keywords:** Regeneration, Mesenchymal stem cells, Induced pluripotent stem cells, Stem-cell research

## Abstract

Since an impaired coronary blood supply following myocardial infarction (MI) negatively affects heart function, therapeutic neovascularization is considered one of the major therapeutic strategies for cell-based cardiac repair. Here, to more effectively achieve therapeutic neovascularization in ischemic hearts, we developed a dual stem cell approach for effective vascular regeneration by utilizing two distinct types of stem cells, CD31^+^-endothelial cells derived from human induced pluripotent stem cells (hiPSC-ECs) and engineered human mesenchymal stem cells that continuously secrete stromal derived factor-1α (SDF-eMSCs), to simultaneously promote natal vasculogenesis and angiogenesis, two core mechanisms of neovascularization. To induce more comprehensive vascular regeneration, we intramyocardially injected hiPSC-ECs to produce de novo vessels, possibly via vasculogenesis, and a 3D cardiac patch encapsulating SDF-eMSCs (SDF-eMSC-PA) to enhance angiogenesis through prolonged secretion of paracrine factors, including SDF-1α, was implanted into the epicardium of ischemic hearts. We verified that hiPSC-ECs directly contribute to de novo vessel formation in ischemic hearts, resulting in enhanced cardiac function. In addition, the concomitant implantation of SDF1α-eMSC-PAs substantially improved the survival, retention, and vasculogenic potential of hiPSC-ECs, ultimately achieving more comprehensive neovascularization in the MI hearts. Of note, the newly formed vessels through the dual stem cell approach were significantly larger and more functional than those formed by hiPSC-ECs alone. In conclusion, these results provide compelling evidence that our strategy for effective vascular regeneration can be an effective means to treat ischemic heart disease.

## Heart attack: Stem cells team up to fix blood vessels

A treatment involving two different types of stem cells leads to repairing failed hearts by making new functional blood vessels. Researchers at the City University of Hong Kong and the Catholic University of Korea induced heart attacks in rats before injecting the hearts with endothelial cells derived from human induced pluripotent stem cells, specialized to form blood vessels. These cells successfully induced the formation of new blood vessels in the damaged hearts. The researchers combined this treatment with a cardiac patch containing engineered human adult stem cells, which improved the survival and performance of the endothelial cells. And this dual stem cell treatment resulted in enhanced cardiac function and a higher number of larger and stronger new blood vessels than those produced by the single-cell treatment suggesting an effective way to repair failed hearts.

## Introduction

Ischemic heart diseases (IHDs) are one of the leading causes of morbidity and motility worldwide^1^. In particular, myocardial infarction (MI), a representative form of IHD, excessively damages both cardiac muscles and blood vessels^2^. Since damage to coronary vessels is one of the most critical pathophysiological etiologies in MI, approaches to date have focused on reconstituting functionally perfusable vasculature in the ischemic heart through therapeutic neovascularization^3^. Despite encouraging outcomes from preclinical studies, the results obtained from subsequent clinical trials failed to produce consistent conclusive outcomes. One probable explanation for this discrepancy between preclinical and clinical outcomes is the failure to maintain optimal levels of targeting angiogenic factors in the target area over a certain duration for prolonged angiogenic stimulation, which is technically challenging and considered prohibitively pricy^4^.

It has become more widely accepted that the perfusable vasculature can be reconstructed in the ischemic heart through therapeutic neovascularization^5^. Several previous studies delineated that therapeutic neovascularization generally comprises two distinct processes, angiogenesis and vasculogenesis. Angiogenesis is defined as the growth of pre-existing blood vessels, and vasculogenesis refers to the de novo generation of vessels from endothelial cells (ECs), endothelial progenitor cells (EPCs), or other types of stem cells^6^. Since several previous studies have extensively reported that the induction of angiogenesis is effective for vascular regeneration, considerable efforts have been invested to develop an efficient strategy to promote myocardial angiogenesis to treat ischemic heart disease. For instance, in a number of preclinical settings, the administration of proangiogenic factors such as vascular endothelial growth factor (VEGF), fibroblast growth factor (FGF), and hepatocyte growth factor (HGF) in the forms of recombinant proteins, naked DNA plasmids or modified VEGF RNA has been shown to substantially improve blood vessel formation, myocardial function, and the long-term survival rate, indicating that promoting angiogenesis is an effective therapeutic strategy to treat failing hearts.

While angiogenesis is known to occur during both embryonic and adult life, vasculogenesis is considered to occur only during embryonic development^7^. However, recent evidence suggests that vasculogenesis is not restricted to early embryogenesis, but may also contribute to the pathophysiology of vascular diseases in postnatal life^8^. This concept was stemmed from the observation that both ECs and EPCs co-exist in the circulation and that new blood vessels develop during tumor growth. This identification of circulating EPCs in adult vertebrates newly suggested a functional role for several types of cells derived from bone marrow in postnatal vasculogenesis^9^. Subsequently, several studies reported that transplantation of EPCs, mononuclear cells (MNCs), and mesenchymal stem cells (MSCs) induced therapeutic neovascularization in adult ischemic tissues^10–12^. However, their potential to differentiate into ECs and undergo successive vasculogenesis is suboptimal because their main mechanisms involve paracrine effects rather than de novo vasculogenesis. More recently, ECs derived from human pluripotent stem cells (hPSC-ECs), including human embryonic stem cells (hESCs) and human induced pluripotent stem cells (hiPSCs), have been suggested as a new cell source for vasculogenesis. Although hPSC-ECs exhibited a relatively higher vasculogenic potential than other cells, their ability to induce vasculogenesis is still insufficient and inefficient^13–15^. For example, a recent study applying hPSC-ECs in a hindlimb ischemia model demonstrated that it took several months to form functional blood vessels in ischemic tissue, suggesting the limited potential of hPSC-ECs in vasculogenesis. Consequently, for hPSC-ECs to be used more effectively for therapeutic neovascularization in ischemic tissues, an innovative method to promote vasculogenic potential must be developed.

Therefore, in the current study, we developed a strategy to effectively reconstruct the vasculature in ischemic hearts by utilizing two distinct types of cell sources, CD31^+^ hiPSC-ECs and genetically engineered MSCs derived from human bone marrow that continuously secrete stromal-derived factor-1α (SDF-eMSCs) within a three-dimensional (3D) cardiac patch implanted in the epicardium of MI-induced hearts. We hypothesize that intramyocardially injected hiPSC-ECs would produce de novo vessels via vasculogenesis, whereas epicardially implanted SDF-eMSC patches (SDF-eMSC-PAs) would simultaneously enhance the angiogenesis of host vessels consistent with the secretion of angiogenic paracrine factors, particularly SDF, in MI-induced hearts. Since the basis of native neovascularization is not restricted to angiogenesis but includes postnatal vasculogenesis as well, we reasoned that strategies targeting therapeutic neovascularization should be focused on comprehensively inducing all these processes together to accomplish true vascular regeneration in ischemic hearts. To use SDF-eMSC-PAs as a cell reservoir to secrete several beneficial cytokines, including SDF-1α, we employed a heart-derived extracellular matrix (hdECM) hydrogel to recapitulate the cardiac tissue-specific microenvironment as closely as possible. Subsequently, we demonstrated that our dual cellular approach with hiPSC-ECs and SDF-eMSC-PAs led to a significant improvement in vessel formation and enhanced cardiac function. These results suggest significant therapeutic implications for vascular regeneration in ischemic hearts.

## Materials and methods

### Manufacture of cardiac patches with decellularized heart-derived extracellular matrix

The hdECM was prepared as described previously^16,17^. Briefly, heart tissue from a 6-month-old Korean domestic pig was purchased from a livestock product market with supplier approval. We dissected the left ventricle from the complete porcine heart and cut it into small pieces. The small pieces of heart tissue were soaked in 1% sodium dodecyl sulfate (Affymetrix, CA) solution for 48 h followed by treatment with 1% Triton X-100 solution in PBS (Biosesang, Korea) for 1 h. Next, the decellularized tissues were dipped in PBS for 3 days to remove the residual detergent. Subsequently, the decellularized heart tissues were lyophilized, pulverized in liquid nitrogen and digested in 10 mL of 0.5 M acetic acid solution (Merck Millipore, Billerica, MA) at a final concentration of 3.3 w/v% (330 mg of hdECM powder) with 33 mg of pepsin powder. The digested hdECM solution was filtered through a 40 µm pore mesh, aliquoted in 1 mL, and stored at -20 °C for further experiments. Before the cardiac patches were manufactured, the hdECM solution was adjusted to a neutral pH of 7.4 by adding 10 N NaOH solution while keeping the conical tube in an ice bucket to avoid gelation of hdECM.

### Myocardial infarction model and patch delivery

All animal studies were approved by the Institutional Animal Care and Use Committee (IACUC) of The Catholic University of Korea (Approval number: CUMC-2020-0051-01). The IACUC and Department of Laboratory Animals (DOLA) at Catholic University of Korea, Songeui Campus accredited the Korea Excellence Animal Laboratory Facility from Korea Food and Drug Administration in 2017 and acquired AAALAC International full accreditation in 2018. All animal procedures conformed to the guidelines from Directive 2010/63/EU of the European Parliament on the protection of animals used for scientific purposes or the NIH guidelines. Fischer 344 rats (160–180 g, male, Koatec, Korea) were anesthetized with 2% inhaled isoflurane and intubated via the trachea with an 18-gauge intravenous catheter. The rats were then mechanically ventilated with a rodent respirator (55-7058, Harvard Apparatus, Canada). Animals were placed on a 37 °C heating pad to prevent cooling during the procedure. After shaving the chest and sterilizing with 70% alcohol, we performed a left thoracotomy. MI was induced by tying a suture with sterile polyethylene glycol tubing (22 G) placed into the left anterior descending (LAD) artery for 1 min, and the knot was permanently ligated using a 7-0 prolene suture. To establish baseline left ventricular function, we examined the ejection fraction (EF) after operation day (POD) 7 (inclusion criterion: EF < 45% based on echocardiographic evaluation). The rats were anesthetized again on the next day using isoflurane inhalation, intubated and mechanically ventilated. The animal chest was re-opened, and the pericardium was partially removed from the infarcted heart. Five experimental groups as follows: (1) Sham control, (2) MI control, (3) SDF-eMSC-PA implanted epicardium of MI hearts (PA only, 1 × 10^6^), (4) hiPSC-ECs, intramyocardial injection (EC only, 1 × 10^6^), and (5) combined platform of hiPSC-ECs and SDF-eMSC-PA (EC + PA, 1 × 10^6^ in each). The hiPSC-ECs were injected at two different sites in the border zone of infarcted heart and the 3D cardiac patches (diameter: 1 cm) were implanted directly on the epicardium using three sutures. The chest was closed aseptically, and antibiotics and 0.9% normal saline solution were given. All rats received the following immunosuppressants as described previously:^18,19^ azathioprine, 2 mg/kg; cyclosporine A, 5 mg/kg; and methylprednisolone, 5 mg/kg daily.

### Echocardiography

The animals were lightly anesthetized with isoflurane and maintained at 37 °C using a heating pad. The assessment of functional improvement in the injured cardiac tissues was performed with echocardiography as we previously described^20,21^. Rats were lightly anesthetized with inhaled isoflurane and physiological data were recorded using a transthoracic echocardiography system equipped with a 15 MHz L15-7io linear transducer (Affniti 50 G, Philips). Serial echocardiograms were performed at pre, 2, 4, and 8 weeks after cell treatment. The echocardiography operator was blinded to the group allocation during the experiment.

### Hemodynamic measurements

Hemodynamic measurements were performed at 8 weeks before euthanasia. After thoracotomy without bleeding, the LV apex of the heart was punctured with a 26 gauge needle, and a 2 F conductance catheter (SPR-838, Millar) was inserted into the LV. LV pressure-volume (PV) parameters were continually recorded using a PV conductance system (MPVS Ultra, EMKA Technologies, Paris, France) coupled to a digital converter (PowerLab 16/35, ADInstruments, Colorado Springs, CO). Load-independent measurements of cardiac function, including the slopes of the end-systolic pressure volume relationship (ESPVR) and end-diastolic pressure volume relationship (EDPVR), were obtained with different preloads, which were elicited via transient inferior vena cava (IVC) occlusion with a needle holder. An aliquot of 50 µl of hypertonic saline (20% NaCl) was injected into the left jugular vein to calculate the parallel conductance after hemodynamic measurements. The blood was collected from the left ventricle into a heparinized syringe and placed into cuvettes to convert the conductance signal to volume using the catheter. The absolute volume of the rat was defined by calibrating the parallel conductance and the cuvette conductance.

### Determination of fibrosis

For determination of the circumferential fibrosis and viable myocardium, Masson’s trichrome staining (Sigma, St. Louis, MO, USA) was performed as follows. Briefly, three paraffin slides were preincubated in a 37 °C dry oven before deparaffinization and rehydration. The paraffin sections were then refixed for one hour in 56 °C Bouin’s solution. These sections were stained using Weigert’s iron hematoxylin solution for 15 min at room temperature and further stained with Biebrich scarlet-acid fuchsin solution for 20 min at room temperature. Finally, the sections were counterstained with aniline blue for 15 min, followed by 1% acetic acid incubation for 1 min at room temperature. Extensive washes were performed between each step. Subsequently, imaging of the heart sections was performed with a slide scanner (Pannoramic MIDI). The ratio of fibrosis was calculated as the area of fibrosis to the area of LV circumference [(infarct area/LV wall area) * 100]. In addition, the ratio of viable myocardium was quantified as the area of viable myocardium within the infarct area where the LV wall is dilated, and the fibrotic area is exhibited. [(viable myocardium area/infarct area) * 100]. Both measurements were performed by using ImageJ software.

### Statistical analysis

All quantitative data are presented as the mean ± S.D unless otherwise indicated. The significant differences between two groups were analyzed by two-tailed Student’s *t* tests. The significant differences between the 4 groups were also analyzed by ANOVA with Bonferroni’s post-hoc analysis. The results were considered statistically significant when the *p* value was less than 0.05.

## Results

### Generation of CD31^+^ endothelial cells derived from hiPSCs and their in vitro characterization

Several previous studies reported successful generation of ECs from hiPSCs (hiPSC-ECs) using a combination of small molecules, including a GSK3 inhibitor^22^. Based on previous reports, we generated hiPSC-ECs from hiPSCs using the GSK3 inhibitor CHIR99021 (Supplementary materials and methods, Supplementary Fig. 1a). To produce hiPSC-ECs expressing green fluorescence protein (GFP) to facilitate cell tracking in the heart tissues in further experiments, we produced hiPSCs expressing GFP signals by transfecting GFP lentiviral particles, enriched them by FACS based on GFP expression, and used them to differentiate into ECs (Supplementary Fig. 1b). qRT-PCR results verified that the expression level of OCT4, a pluripotency marker, was significantly reduced, and the expression level of CD31, a specific marker for EC, was significantly increased in the hiPSCs differentiating into the EC lineage (Supplementary Fig. 1c, Supplementary Table 1). On differentiation Day 7, we observed that approximately 25.08% of the differentiating hiPSC-ECs were positive for human CD31 antibody, and subsequently, we enriched these CD31^+^ cells by FACS. Following FACS, the enriched CD31^+^ hiPSC-ECs were maintained in human endothelial serum-free medium with cytokines, including VEGF, to maintain their characteristics as EC lineage cells (Supplementary Fig. 1c). The CD31^+^ hiPSC-ECs displayed a typical cobblestone-like EC morphology and expressed similar mRNA levels of EC-specific markers, such as *cluster of differentiation 31 (CD31), vascular endothelial cadherin (VE-Cadherin), Von Willebrand factor (vWF)* and *vascular endothelial growth factor receptor 2 (VEGFR2)*, compared with human umbilical cord endothelial cells (HUVECs) (Supplementary Fig. 1c, d). The results from flow cytometry analysis further demonstrated that the CD31^+^ hiPSC-ECs were 97.19% and 85.53% positive for CD31 and CD144, respectively (Supplementary Fig. 1e). In addition, the immunofluorescence results confirmed that the CD31^+^ hiPSC-ECs expressed abundant levels of the CD31 and vWF proteins (Supplementary Fig. 1f). At the functional level, the CD31^+^ hiPSC-ECs displayed the capacity for uptake of Ac-LDL (Supplementary Fig. 1g) and the formation of a capillary-like network on top of Matrigel (Supplementary Fig. 1h).

### Intramyocardially injected hiPSC-ECs generate de novo vessels in MI-induced hearts

**Fig. 1:**
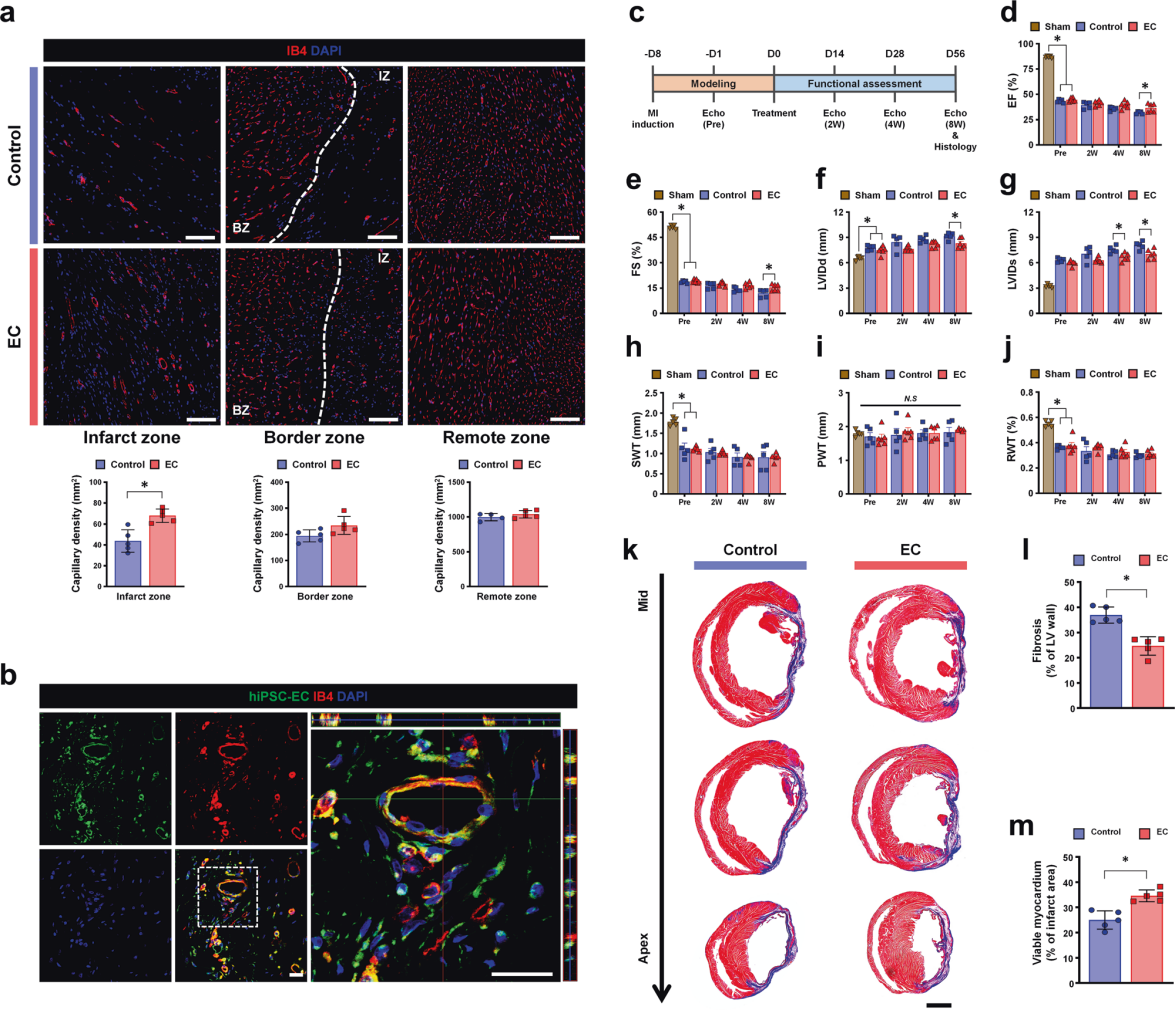
Intramyocardial injection of hiPSC-ECs improves the formation of de novo vessels and cardiac function in MI-induced hearts. **a** Representative images of blood vessels stained with IB4-rhodamine (red) in the infarct zone, border zone, and remote zone and at 8 weeks after injection of hiPSC-ECs and their quantification summary. For quantification, the number of capillaries in five randomly selected fields (mm^2^) in each heart was counted. *n* = 5. *p < 0.05. Scale bars: 100 µm. **b** Representative image of blood vessels newly formed by iPSC-ECs-GFP (green), IB4-rhodamine (red) and DAPI (blue). Scale bars: 20 µm. **c–j** Rats undergoing MI were intramyocardially injected with hiPSC-ECs or control cells, followed by echocardiography analysis. **c** The schematic timeline from MI modeling and transplantation of iPSC-EC to measurement of cardiac function. **d** Left ventricular ejection fraction (EF), (**e**) left fractional shortening (FS), (**f**) left ventricular internal diastolic dimension (LVIDd), (**g**) left ventricular internal systolic dimension (LVIDs), (**h**) septal wall thickness (SWT), (**i**) posterior wall thickness (PWT), and (**j**) relative wall thickness (RWT). *n* = 6. *p < 0.05. **k** Representative images showing cardiac fibrosis after staining with Masson’s trichrome in the hearts harvested 8 weeks after cell treatment. Quantification results of cardiac fibrosis (**l**) and viable myocardium (**m**). *n* = 5. *p < 0.05. Scale bars: 2000 µm.

To determine if the CD31^+^ hiPSC-ECs (hiPSC-ECs afterward) could form de novo vessels via a vasculogenesis-dependent mechanism in MI-induced hearts, we intramyocardially injected hiPSC-ECs at two different sites in the border zone of the MI-induced rat hearts. MI was generated by ligation of the left anterior descending (LAD) artery in the heart. hiPSC-ECs continuously expressing the green fluorescence (GFP) signal were used for tracking purposes. To visualize the functional vessels in the MI-induced hearts, we performed perfusion staining with isolection-B4 (IB4) conjugated with a red fluorescent dye, rhodamine, into the heart prior to tissue harvest 8 weeks after injection with hiPSC-ECs. Fluorescent image analyses showed that the number of IB4^+^ capillaries in the hiPSC-EC-injected hearts was significantly higher than that in the MI control hearts (Fig. 1a).

Next, to evaluate the potential and magnitude of the contribution of hiPSC-ECs to vasculogenesis in the MI hearts, we traced the GFP and RFP signals from hiPSC-ECs within cardiac tissues. Confocal microscopy images demonstrated a considerable number of vessels, double-positive for both IB4 and GFP signals from hiPSC-ECs, in the infarct region of the heart tissues receiving hiPSC-ECs at 8 weeks post-injection. Interestingly, a substantial number of hiPSC-ECs were incorporated into the host capillary network, and many of them were located in the perivascular area (Fig. 1b). The results clearly suggest that hiPSC-ECs could reconstruct de novo vessels in ischemic hearts.

Given that vascular regeneration improved through vasculogenesis leads to functional recovery from MI, we hypothesized that intramyocardial injection of hiPSC-ECs into MI hearts may promote cardiac function. Subsequently, we performed serial echocardiography to evaluate left ventricular (LV) function and cardiac remodeling from PRE (1-week post-MI and prior to cell treatment), and 2, 4, and 8 weeks after cell treatment. In this study, we employed a MI model that cells were transplanted one week after induction of MI to mimic the clinical situation of MI patients as close as possible. The results of echocardiography demonstrated that both ejection fraction (EF) and fractional shortening (FS) in all experimental groups were significantly lower compared with the sham group that did not receive any intervention. (Supplementary Fig. 2a–g). Of importance, the hearts receiving hiPSC-ECs displayed significantly higher EF and FS than those in the MI control group until 8 weeks after the cell treatment (Fig. 1c–d). Among several parameters for cardiac remodeling, such as left ventricular internal diastolic dimension (LVIDd), left ventricular internal systolic dimension (LVISd), septal wall thickness (SWT), posterior wall thickness (PWT), and relative wall thickness (RWT), the LVIDd and LVIDs in the hiPSC-EC-treated hearts were significantly lower than those in MI control hearts, indicating that hiPSC-ECs protected the hearts from adverse cardiac remodeling. (Fig. 1e–i and Supplementary Fig. 2h). Similarly, the results of Masson’s trichrome staining obtained using cardiac tissue harvested at 8 weeks post-cell treatment showed that the area of fibrosis (%) in the hiPSC-EC-injected group was considerably smaller and the viable myocardium (%) was larger than that in the MI control group (Fig. 1j–m). Based on these results, we confirmed that hiPSC-ECs can directly contribute to de novo vessel formation in vivo in MI-exposed hearts, resulting in enhanced cardiac function.

### Cellular characteristics of SDF-eMSCs

Subsequently, we investigated our central hypothesis of whether simultaneous induction of both vasculogenesis and angiogenesis could lead to comprehensive vascular regeneration and functional improvement in the MIhearts. Since we already verified that hiPSC-ECs successfully achieved vasculogenesis in the MI hearts, we sought to identify an additional cellular source that can induce complementary angiogenesis from the blood vessels in the host heart and finally decided to test genetically modified human mesenchymal stem cells engineered to continuously release human SDF-1α protein (SDF-eMSCs)^23^. The SDF-eMSCs were indistinguishable from normal BM-MSCs. The SDF-eMSCs exhibited a homogeneous spindle-shaped cell morphology, representing hMSCs (Supplementary materials and methods, Supplementary Fig. 3a). The SDF-eMSCs had a high proliferative potential based on the gradual increase in population doubling levels (PDL) during the culture times compared to normal BM-MSCs^24^ (Supplementary Fig. 3b). The SDF-eMSCs expressed several markers specific for human MSCs, such as CD90, CD44, CD105 and CD73, without the expression of CD34, CD11b, CD19, CD45 and HLA-DR (Supplementary Fig. 3c). The SDF-eMSCs stably secreted human SDF-1α protein, as determined by human SDF-1α enzyme-linked immunosorbent assay (ELISA) analysis (Supplementary Fig. 3d). The results from SDF-eMSC karyotyping revealed a normal human karyotype of the SDF-eMSCs without chromosomal abnormalities, suggesting the genetic stability of the SDF-eMSCs (Supplementary Fig. 3e).

### SDF-eMSCs enhance the angiogenic potential of endothelial cells in vitro

**Fig. 2:**
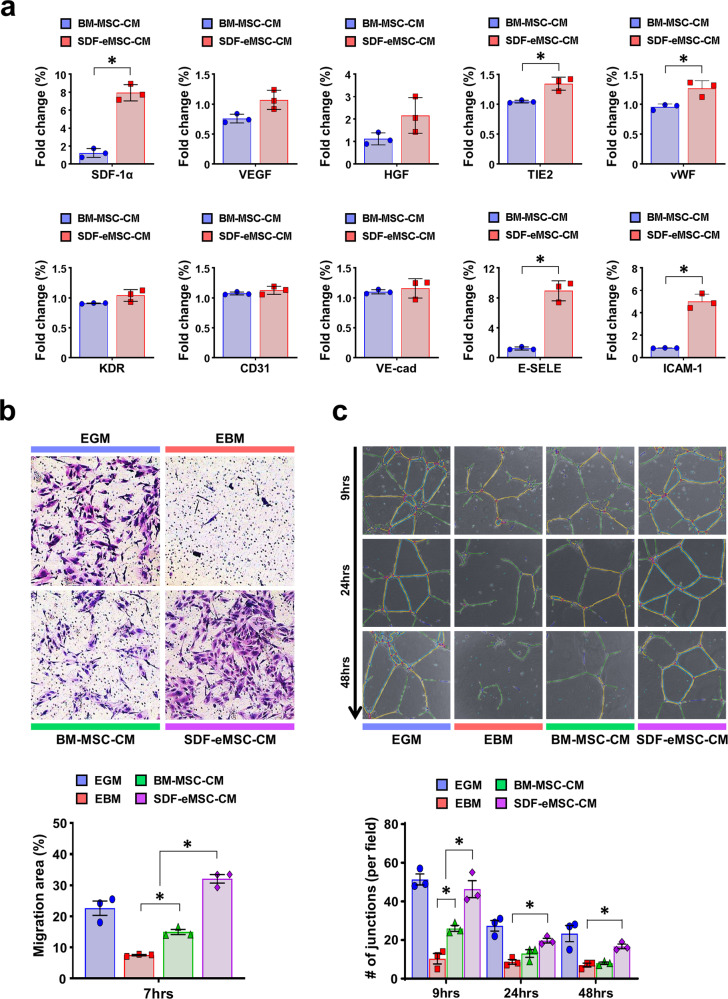
SDF-eMSCs enhance the angiogenic potential of hiPSC-ECs in vitro. **a** qRT-PCR analysis of relative mRNA expression associated with ECs and angiogenesis in the hiPSC-ECs treated with the conditioned media (CM) from cultured bone marrow mesenchymal stem cells (BM-MSC-CM) or SDF engineered MSCs (SDF-eMSC-CM) for 3 days. The y-axis represents the relative mRNA expression of target genes to glyceraldehyde-3-phosphate dehydrogenase (GAPDH). *n* = 3. *p < 0.05. **b** EC migration assay. Representative images of migrated hiPSC-ECs and quantification of the migrated area (%). The hiPSC-ECs were placed in transwells (top), and regular media (EGM, EBM) or the conditioned media (CM) collected from different cell sources (BM-MSC-CM and SDF-eMSC-CM) were placed in transwells (bottom) for 7 h. *n* = 3. *p < 0.05. **c** Tube formation assay. The hiPSC-ECs were cultured in 24-well plates coated with Geltrex™ with regular media (EGM, EBM) or conditioned media (CM) (BM-MSC-CM and SDF-eMSC-CM) for 9, 24, or 48 h. Representative images of tube formation and quantification summary for the number of junctions. *n* = 3. *p < 0.05.

To investigate whether SDF-eMSCs could augment the angiogenic potential of ECs, we performed various types of in vitro experimental analyses with SDF-eMSCs. Among the first, to determine whether SDF-eMSCs influenced the gene expression associated with ECs and angiogenic properties, we treated 30% conditioned media (CM) harvested from cultured SDF-eMSCs or BM-MSCs to the cultured hiPSC-ECs for 3 days and performed qRT-PCR analyses. The expression levels of *stromal-derived factor-1 alpha (SDF-1α), tyrosine kinase with Ig and epidermal growth factor homology domain 2* (*Tie-2*)*, vWF, E-selectin* (*CD62*)*, and intercellular adhesion molecule-1* (*ICAM-1*) were significantly higher in the hiPSC-ECs treated with SDF-eMSC-CM than in the hiPSC-ECs exposed to BM-MSC-CM (Fig. 2a). In particular, the increased expression of *E-selectin* and *ICAM-1* is known to be involved in angiogenesis in the presence of activated ECs^25–29^. Next, in EC migration assays, as shown in Fig. 2, the addition of conditioned media from the SDF-eMSCs (SDF-eMSC-CM) significantly enhanced the migration of hiPSC-ECs or HUVECs compared with the migration of the ECs treated with CM from human bone marrow-derived MSCs (BM-MSC-CM), suggesting that cytokines released from the SDF-eMSCs bolster the mobility of ECs (Fig. 2b and Supplementary Fig. 4a). In addition, to test whether SDF-eMSCs directly promote the angiogenic potential of ECs, we performed Matrigel tube formation assays, a representative experiment to evaluate the vessel formation potential of cells. The results from Matrigel tube formation assays demonstrated that the number of branches formed in both the hiPSC-ECs and the HUVECs treated with 30% CM harvested from the cultured SDF-eMSCs was significantly greater than that in the BM-MSC-CM-treated ECs (Fig. 2c and Supplementary Fig. 4b). Interestingly, treatment with SDF-eMSC-CM not only promoted tube formation by the hiPSC-ECs but also contributed to the maintenance of vessels formed from the hiPSC-ECs. Unlike the hiPSC-EC-generated vessels exposed to BM-MSC-CM that began to disrupt the vessel structure within 24 h of vessel formation, treatment with SDF-eMSC-CM supported the integrity of vessels for up to 48 h.

### SDF-eMSC-PAs consistently secrete SDF-1α

**Fig. 3:**
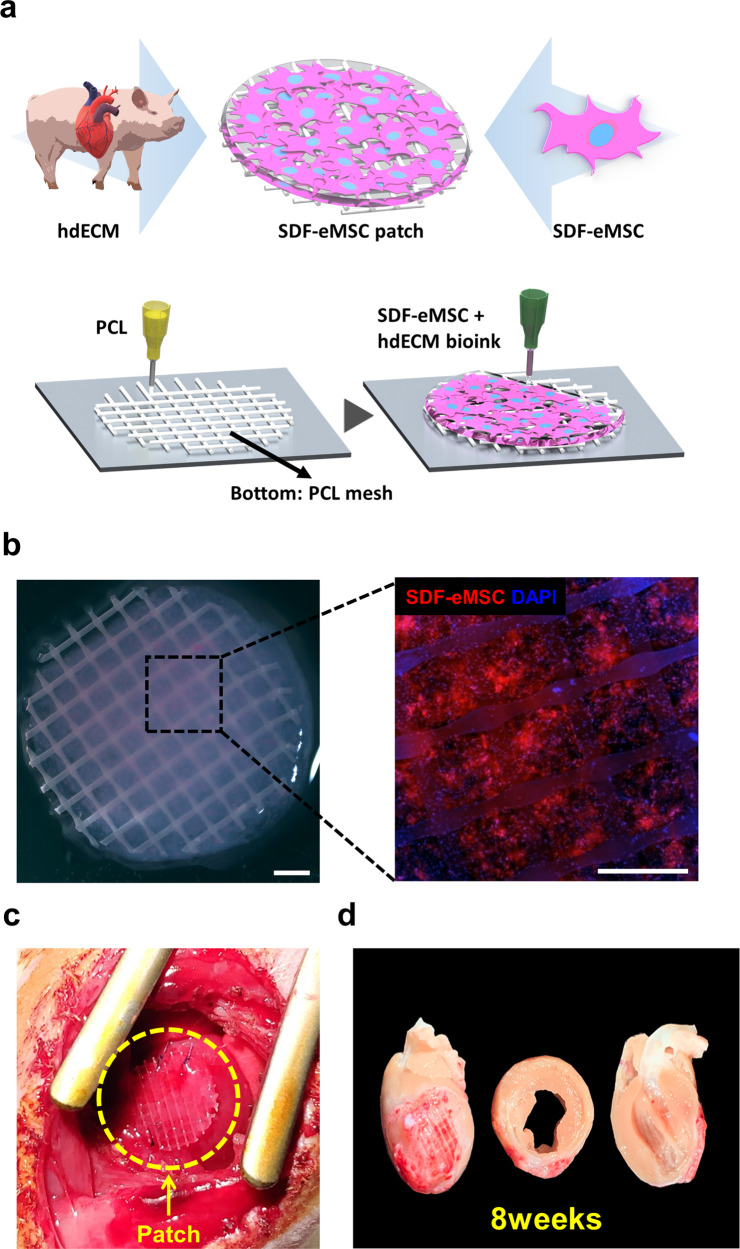
Schematic illustration of SDF-eMSC-PA production using hdECM. **a** Procedures for manufacturing a cardiac patch encapsulating SDF-engineered MSCs (SDF-eMSC-PA) with a polycaprolactone (PCL) platform produced by a 3D printing system. **b** Optical image within the hdECM patch. SDF-eMSC-PAs were prelabeled with the red florescence dye DiI for tracing. Scale bars: 1 mm. **c** Image of epicardially transplanted SDF-eMSC-PAs in the MI-induced heart. **d** Macroscopic view of hearts at 8 weeks after PA transplantation.

To provide a cellular reservoir where SDF-eMSC-PAs can constantly release SDF-1α to MI hearts, we produced a patch encapsulating SDF-eMSC (SDF-eMSC-PA) by mixing SDF-eMSCs with a 2% heart-derived decellularized extracellular matrix (hdECM)-based bioink and loaded it onto the polycaprolactone (PCL) mesh (Fig. 3 and Supplementary Fig. 5). Subsequently, to confirm whether SDF-eMSC-PAs are functional and can efficiently release SDF-1α, we cultured SDF-eMSC-PAs in vitro for 28 days (Supplementary Fig. 5a) and collected supernatants at various time points for three days to generate the release kinetics of the SDF1α-eMSC-PAs using the SDF-1α ELISA kit. The cumulative release curve showed that although the initial concentration of SDF-1α was higher in the SDF-cytokine-PAs (300 ng/ml) than in the SDF-eMSC-PAs on Day 0, no SDF-1α was detectable in the SDF-cytokine-PAs from Day 7. However, the expression of SDF-1α released from the SDF-eMSC-PAs increased consistently until Day 21 (Supplementary Fig. 5b), suggesting that SDF-eMSCs continuously secreted SDF-1α within the patch.

### Combined treatment with hiPSC-ECs and SDF-eMSC-PAs improved cardiac function and reduced scar formation following MI

**Fig. 4:**
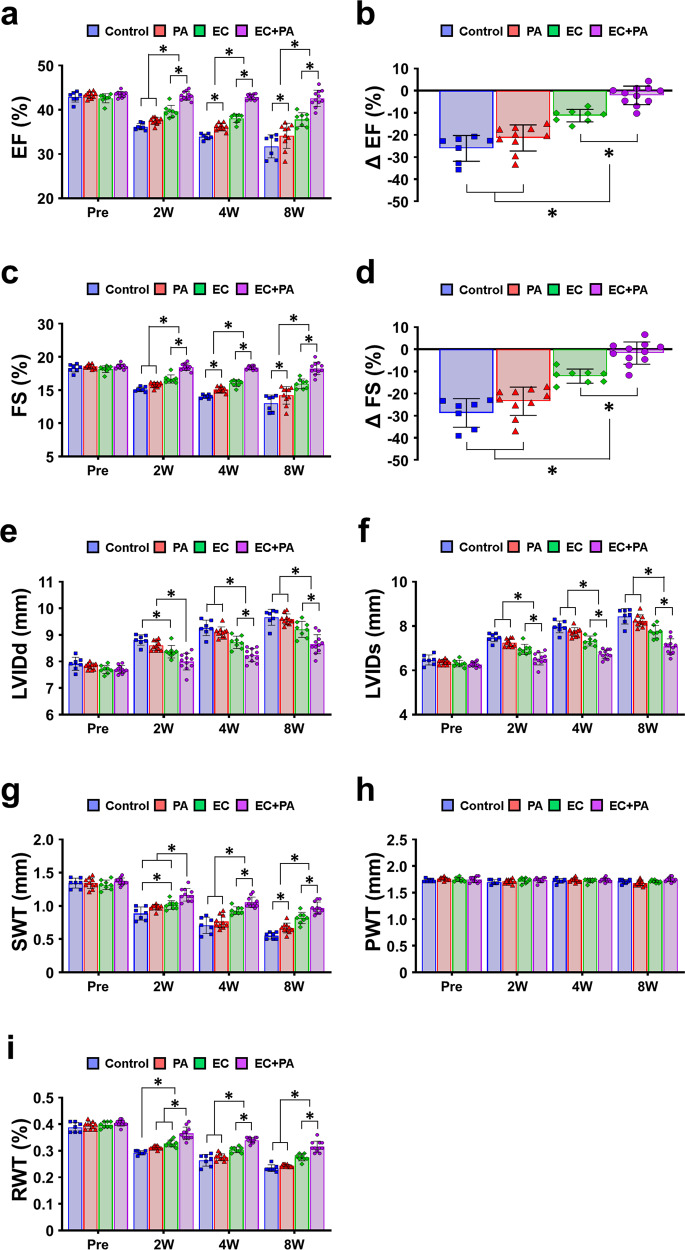
The combined platform with hiPSC-ECs and SDF-eMSC-PAs significantly amplifies cardiac function in infarcted hearts. **a** Left ventricular ejection fraction (EF). **b** EF delta change at 8 weeks after cell treatment. **c** Left fractional shortening (FS). **d** FS delta change at 8 weeks after cell treatment. **e** Left ventricular internal diastolic dimension (LVIDd). **f** Left ventricular internal systolic dimension (LVIDs). **g** Septal wall thickness (SWT). **h** Posterior wall thickness (PWT). **i** Relative wall thickness (RWT). *n* = 6–11. *p < 0.05.

**Fig. 5:**
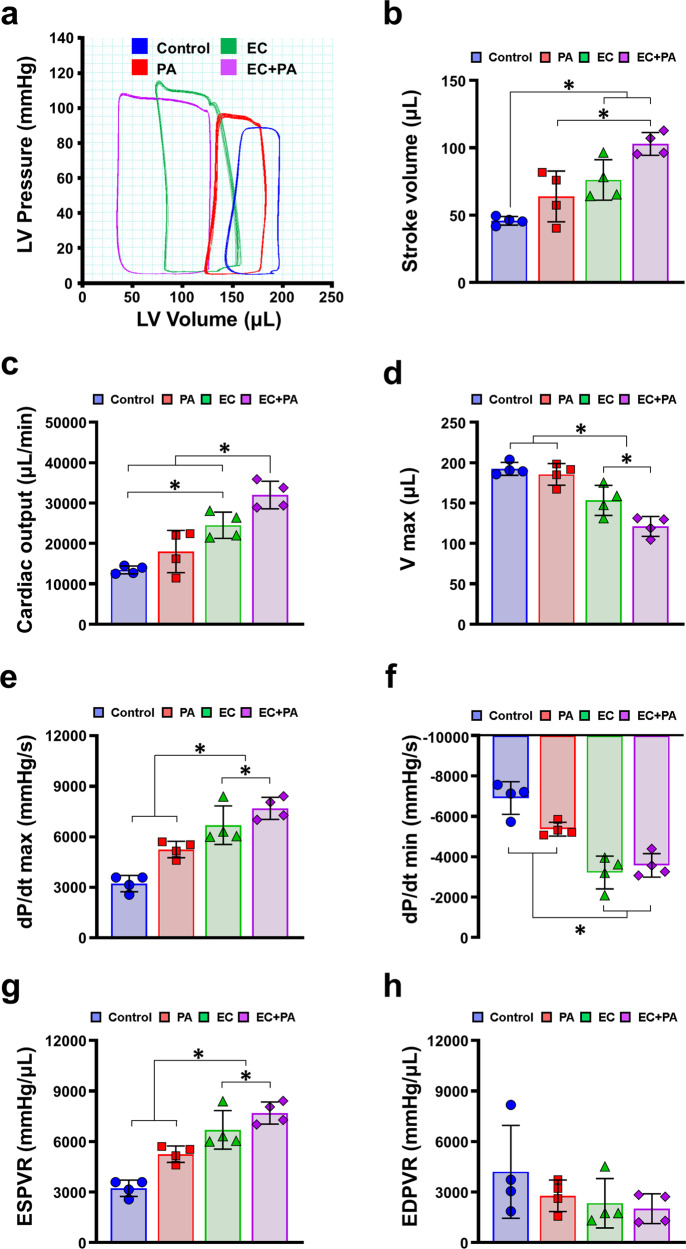
The combined platform with hiPSC-ECs and SDF-eMSC-PA improves cardiac contractibility. **a** Representative images of the hemodynamic pressure and volume (PV) curve at steady state at 8 weeks post-cell treatment. **b** Stroke volume (SV). **c** Cardiac output (CO). **d** Volume max (V max) defining the amount of blood volume in the LV at end-diastole. **e** dP/dt_max_ refers to the maximal rate of pressure changes during systole. **f** The minimal rate of pressure changes during diastole (dP/dt_min_). **g** Slope of end-systolic pressure volume relationship (ESPVR) indicating the intrinsic cardiac contractibility as measured by transient inferior vena cava (IVC) occlusion. **h** Slope of end-diastolic pressure volume relationship (EDPVR). *n* = 4. *p < 0.05.

To finally determine whether simultaneous induction of both vasculogenesis and angiogenesis by using hiPSC-ECs and SDF-eMSC-PAs could lead to comprehensive vascular regeneration and functional improvement in MI-induced hearts, we induced MI by LAD ligation after the formation of five experimental groups as follows: (1) MI control, (2) SDF-eMSC-PA implanted epicardium of MI hearts (PA only, 1 × 10^6^), (3) hiPSC-ECs, intramyocardial injection (EC only, 1 × 10^6^), and (4) combined platform of hiPSC-ECs and SDF-eMSC-PA (EC + PA, 1 × 10^6^ in each) (Fig. 4). We first performed serial echocardiography for all experimental groups at pre, 2, 4 and 8 weeks after cell treatment. All experimental groups were significantly reduced compared with that in the sham group (Supplementary Fig. 6a–g). Of interest, cardiac function in the EC + PA group was significantly preserved until 8 weeks compared with its cardiac function at pre, but cardiac function in other groups, such as the control, hiPSC-EC alone and SDF-eMSC-PA alone groups, continuously decreased until 8 weeks. (Fig. 4a–d). Adverse cardiac remodeling determined by the LVIDd, LVIDs, SWT, PWT, and RWT was notably reduced in the EC + PA group compared with the other groups (Fig. 4e–i and Supplementary Fig. 6h). To further evaluate cardiac function more precisely, we performed LV hemodynamic measurements using an invasive pressure-volume (PV) catheter, which can measure the hemodynamic pressure and volume of the LV. The results of the PV loop at 8 weeks post-cell treatment showed that the EC + PA group had significantly improved cardiac function and prevented adverse cardiac remodeling compared with the other groups (Fig. 5). The two parameters of general cardiac function, stroke volume (SV) and cardiac output (CO), were significantly higher (Fig. 5a–c), and the maximum volume (V max), which is the cardiac remodeling index measured at the maximum diastole, was significantly lower in the EC + PA group than in the other groups (Fig. 5d). Although the pressure max (P max) measured at the maximum systole did not differ significantly between groups, the maximum rate of pressure change (dP/dt_max_) and the minimum rate of pressure change (dP/dt_min_), which indicate the pressure change in LV per second, were increased in the EC + PA group. (Fig. 5e–f and Supplementary Fig. 7a). Temporal variation in the occluded inferior vena cava (IVC) was used to evaluate load-independent intrinsic cardiac contractibility. The end-diastolic pressure-volume relationship (EDPVR), which indicates the absence of diastolic dysfunction, did not differ between the groups, whereas the slope of the end-systolic pressure volume relationship (ESPVR), which indicates cardiac contractibility, was significantly improved in the EC + PA group compared with the other groups (Fig. 5g–h and Supplementary Fig. 7b). Collectively, these results from LV hemodynamic measurements consistently demonstrate that treatment with the combined platform with hiPSC-ECs and SDF-eMSC-PAs improves cardiac repair in MI hearts.

### A combined approach enhances the engraftment of intramyocardially injected hiPSC-ECs and improves therapeutic neovascularization

**Fig. 6:**
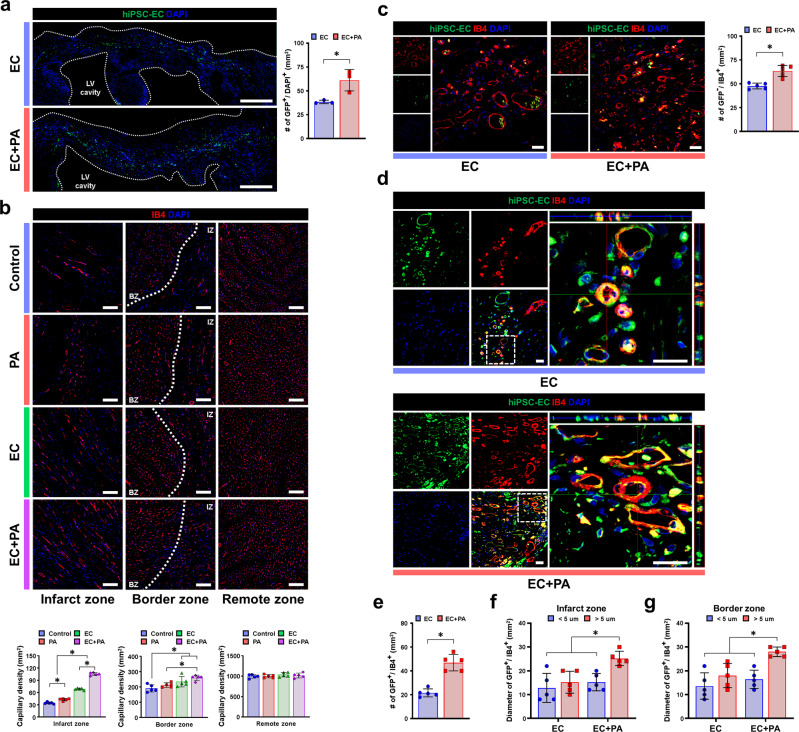
The combined approach with hiPSC-ECs and SDF-eMSC-PA enhances the retention of intramyocardially injected hiPSC-ECs and improves therapeutic neovascularization. **a** Representative image of hiPSC-ECs-GFP within the infarct area at 8 weeks post-cell treatment and their quantification summary. *n* = 3. *p < 0.05. Scale bars: 1000 µm. **b** Representative images of blood vessels stained with IB4-rhodamine (red) in the infarct zone (IZ), border zone (BZ), and remote zone at 8 weeks after cell treatment and a summary of their quantification. *n* = 5–7. *p < 0.05. Scale bars: 100 µm. **c** Representative images of blood vessels negative for GFP but positive for IB4 (GFP^-^/IB4^+^) in the infarcted area and their quantification summary. hiPSC-ECs-GFP (green), IB4-rhodamine (red) and DAPI (blue). *n* = 5. *p < 0.05. Scale bars: 20 µm. **d**, **e** Representative images of GFP and IB4 (GFP^+^/IB4^+^)-positive blood vessels in the infarcted area and their quantification. hiPSC-ECs-GFP (green), IB4-rhodamine (red) and DAPI (blue). *n* = 5. *p < 0.05. Scale bars: 20 µm. **f**, **g** Diameter of hiPSC-EC-derived GFP-positive blood vessels in the infarcted area and border zone. *n* = 5. *p < 0.05.

Next, we investigated the in vivo behavior of intramyocardially implanted hiPSC-ECs in the presence or absence of SDF-eMSC-PAs. Since hiPSC-ECs constantly express GFP, we could track their fate in heart tissue sections. Confocal microscopic examination of heart tissues harvested at 8 weeks after cell treatment demonstrated that implantation of SDF-eMSC-PAs significantly improved the retention and engraftment of intramyocardially injected GFP-positive hiPSC-ECs. Quantitatively, the proportion of GFP-positive hiPSC-ECs in the EC + PA group was substantially higher than that in the EC group (Fig. 6a). Of interest, while the hiPSC-ECs in the hiPSC-ECs alone group were localized near the injection sites, the hiPSC-ECs in the EC + PA group were distributed throughout the regions of the left ventricle. Given the ability of SDF-eMSC-PAs to improve the survival and retention of injected hiPSC-ECs, we sought to examine whether SDF-eMSC-PAs exerted direct cytoprotective effects in hiPSC-ECs in vitro. Ischemic injury was simulated by exposing hiPSC-ECs to H_2_O_2_ (500 µM). Administration of CM from SDF-eMSCs (SDF-eMSC-CM) significantly improved the viability of both hiPSC-ECs and HUVECs as determined by the LIVE/DEAD assay and CCK-8 assay (Supplementary Fig. 8a–f). Treatment with SDF-eMSC-CM substantially increased the number of viable cells, suggesting that SDF-eMSC-CM exerts direct cytoprotective effects on ECs against ischemic insults. Subsequently, we performed thorough histological analyses using heart tissues harvested 8 weeks post-cell treatment to examine whether SDF-eMSC-PAs could concurrently promote hPSC-EC-dependent vasculogenesis as well as angiogenesis of host blood vessels. IB4 conjugated with rhodamine was systemically injected to identify the functional endothelium in these experiments. Initially, confocal images demonstrated that the number of total IB4-positive (IB4^+^) capillaries in both the border zone and infarct zone of the hearts in the EC + PA group was substantially higher than that in the other groups, including the EC group (Fig. 6b). The number of vessels that were GFP negative but positive for IB4 (GFP^-^/IB4^+^) was also significantly higher than that in other groups, including the EC-only group (Fig. 6c). These results suggest that the combined approach significantly promoted the angiogenesis of host vessels in MI hearts. More importantly, the number of de novo vessels formed by hiPSC-ECs-GFP^+^ was substantially higher in the EC + PA group than in the EC-only group, indicating that SDF-eMSC-PAs facilitates hiPSC-EC-dependent vasculogenesis (Fig. 6d–e and Supplementary Fig. 9a). Notably, the number of larger blood vessels (diameter range: >5 μm), one of the indicators of functional blood vessels in the EC + PA group, was significantly higher than that in the EC group. Of interest, many of those larger vessels in the EC + PA group displayed abundant expression of α-SMA, a marker for smooth muscle cells, suggesting that these larger vessels (CD31^+^/α-SMA^+^) may be arteriole-like vessels, indicating that SDF-eMSC-PA played certain roles in vascular ingrowth and maturation (Fig. 6e–g and Supplementary Fig. 9b).

### A combined approach protects the myocardium from ischemic injury and reduces cardiac fibrosis

**Fig. 7:**
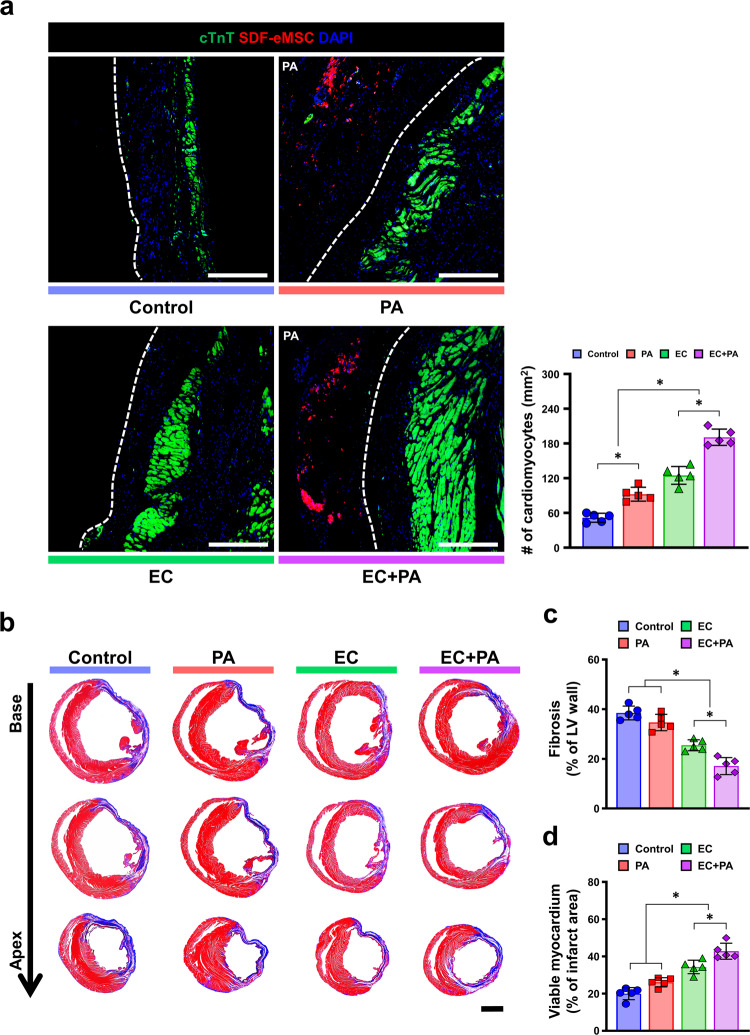
The combined approach protects the myocardium from ischemic injury and reduces cardiac fibrosis. **a** Representative immunostaining images of myocardium stained with cTnT (green) and DAPI (blue) at 8 weeks after cell treatment and quantification of the number of cTnT-positive cardiomyocytes. SDF-eMSCs labeled with DiI within the cardiac patch (red) *n* = 5. *p < 0.05. Scale bar: 300 µm. **b** Representative images of Masson’s trichrome staining using heart tissues harvested 8 weeks after cell treatment. **c**, **d** Quantification summary of a percentage of fibrosis and viable myocardium. *n* = 5. *p < 0.05. Scale bars: 2000 µm.

To further investigate whether the vascular regeneration achieved by the combined platform (EC + PA) was sufficient to salvage the myocardium from ischemic insult, we quantified the viable myocardium by immunostaining for cardiac troponin T (cTnT) antibody using the heart tissues harvested from all experimental groups at 8 weeks post-cell treatment. The number of viable cTnT^+^ cardiomyocytes in the EC + PA group was significantly higher than that in the other groups (Fig. 7a). These results from histological analyses using heart tissues motivated us to test whether SDF-eMSCs (Supplementary Fig. 10a) could confer direct cytoprotective effects on cardiomyocytes against ischemic insults in vitro. Ischemic injury was simulated by exposing cardiomyocytes to H_2_O_2_ (500 µM). The results from both the LIVE/DEAD assay and the cholecystokinin-8 (CCK-8) assay demonstrated that the administration of SDF-eMSC-conditioned media (CM) significantly improved the viability of cultured cardiomyocytes isolated from neonatal rats (NRCM) against H_2_O_2_ treatment compared with other treatments. These results also suggest that SDF-eMSCs have direct cytoprotective effects against ischemic insults (Supplementary Fig. 11a–c).

Consequently, the combined treatment group showed a significant decrease in cardiac fibrosis. The results of Masson’s trichrome staining using cardiac tissue harvested at 8 weeks exhibited an area of fibrosis (%), which was significantly lower in the combined treatment groups than in the other groups (Fig. 7b–d). Taken together, our results clearly suggested that the combined treatment resulted in comprehensive cardiac repair through enhanced vascular regeneration and that the SDF-eMSCs contributed at least to some extent indirect protection of myocardium from ischemic injury via consistent secretion of cytoprotective SDF cytokines.

## Discussion

**Fig. 8:**
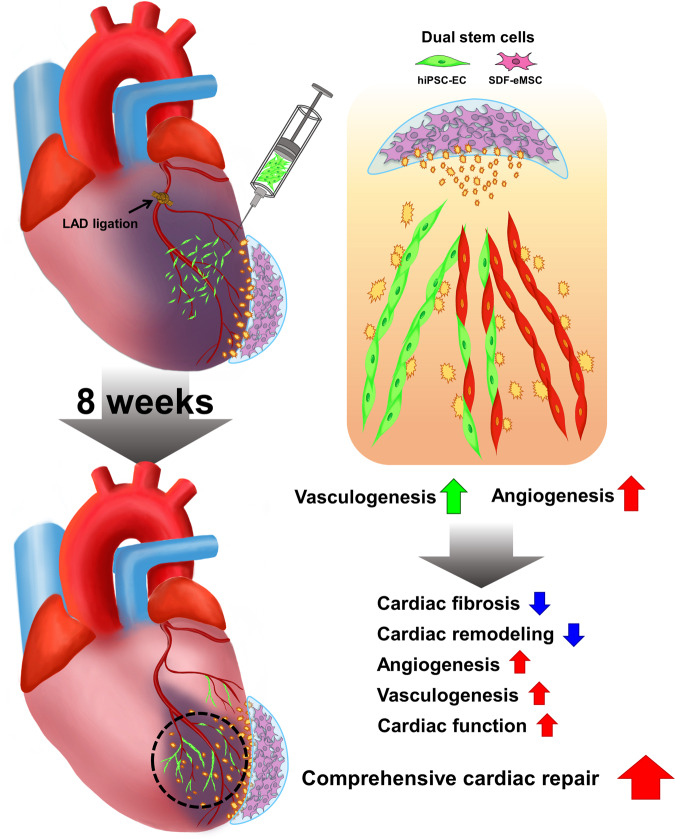
Schematic diagram illustrating the therapeutic mechanisms. The improvement of cardiac function and the comprehensive vascular regeneration achieved by the combined platform with hiPSC-ECs and SDF-eMSC-PAs.

In the present study, we sought to develop a multifaceted strategy to achieve effective therapeutic neovascularization by using hiPSC-ECs and SDF-eMSCs, possibly through simultaneous induction of both postnatal vasculogenesis and angiogenesis (Fig. 8). We observed that intramyocardially injected hiPSC-ECs resulted in the successful formation of de novo vessels via a postnatal vasculogenesis mechanism, whereas epicardially implanted SDF-eMSC-PAs effectively promoted angiogenesis of existing host vessels from the MI heart as well as those newly formed vessels from the injected hiPSC-ECs via continuous secretion of proangiogenic cytokines, particularly SDF-1α.

Apparently, the SDF-eMSC-PAs provided a favorable microenvironment for the intramyocardially injected hiPSC-ECs, which improved their survival, migration, and retention in the ischemic hearts. More importantly, the SDF-eMSC-PAs substantially induced subsequent vascular maturation from the primitive state of de novo vessels formed by the hiPSC-ECs to larger functionally perfusable vessels. Collectively, these synergistic effects of the hiPSC-ECs and SDF-eMSC-PAs ultimately achieved complete vascular regeneration in MI-exposed hearts. Indeed, numerous previous studies have attempted therapeutic neovascularization targeting either vasculogenesis or angiogenesis separately with multiple cell types, including EPCs, hMSCs or cardiac progenitor cells^11,30–33^. Compared with previous studies, this study, to the best of our knowledge, is the first to induce inclusive neovascularization via induction of both vasculogenesis and angiogenesis together by using two distinct major stem cell types that were delivered via two different routes.

To achieve postnatal vasculogenesis in MI hearts, we employed hiPSC-ECs mostly due to their relatively greater vasculogenic potential and similarities with human primary ECs in terms of the expression of endothelial-specific genes and structural proteins as well as physiological characteristics such as tube formation, LDL uptake, and capillary formation in vivo. Several preclinical studies have demonstrated that hPSC-ECs can be used to successfully engraft, align, and couple with host capillaries to increase vascularity^34,35^. More importantly, hPSC-ECs restored perfusion in hindlimb ischemia models and accelerated wound healing, thereby representing an ideal source of cells to treat vascular diseases^36–38^. Although it has been suggested that hPSC-ECs possess comparatively higher vasculogenic potential than other cell types investigated previously, the current study and others consistently demonstrate that treatment with only hPSC-ECs may be insufficient to generate an adequate number of new blood vessels in the ischemic heart owing to the extremely hostile environment in the MI heart suggesting the need for additional support. Thus, in order to promote hiPSC-EC-based vasculogenesis and induce angiogenesis from host vessels, we fabricated a cardiac patch by encapsulating SDF-eMSCs genetically engineered to continuously release SDF-1α.

SDF-1α was selected as a major inducer of myocardial angiogenesis because this molecule is closely involved in multiple physiological phenomena in ECs, such as cell proliferation, survival, migration, angiogenesis and antiapoptotic effects^39,40^. Nonetheless, the therapeutic use of SDF-1α is limited by its short half-life^41^. Therefore, we engineered human BM-MSCs to facilitate the continuous release of SDF-1α using a lentiviral vector encoding hTERT and c-Myc reprogramming factors. We demonstrated that treating conditioned media from SDF-eMSCs with the cultured hiPSC-ECs and HUVECs significantly improved their angiogenic potential, such as enhanced expression of several angiogenesis-related genes, EC proliferation, migration and tube formation. To ensure persistent secretion of SDF-1α and other favorable paracrine factors toward the injured myocardium, we generated hdECM-derived cardiac patches to serve as a cellular reservoir within the infarcted area. To engineer the cardiac patches, we developed an innovative strategy involving the preparation of lyophilized porcine hdECM optimized for convenient storage and easy transport of cell-free ECMs mixed with SDF-eMSCs as bioink. Our results showed that the SDF-eMSCs appeared to survive better within the patch until 8 weeks after treatment, as evidenced by the higher number of DiI-positive MSCs. The surviving SDF-eMSCs constantly secreted beneficial paracrine factors and promoted vascular regeneration by promoting hiPSC-EC-dependent vasculogenesis and angiogenesis and ultimately rejuvenated the injured myocardium.

Indeed, significant cell death and poor engraftment rates following transplantation into the host myocardium are some of the most critical hurdles limiting cell-based vascular regeneration in the ischemic heart. Interestingly, our histological analyses revealed a considerable increase in the retention and engraftment of intramyocardially injected hiPSC-ECs, which induced robust and stable vascular regeneration when paired with epicardial SDF-eMSC-PAs. Conversely, the absence of SDF-eMSC-PAs resulted in a rapid decline in the number of hiPSC-ECs over 8 weeks. In contrast, its presence greatly increased the number of surviving hiPSC-ECs for subsequent vessel formation. Thus, the paracrine factors secreted by SDF-eMSC-PAs initially facilitated rapid stabilization of injected hPSC-ECs and improved the survival and engraftment of hiPSC-ECs, particularly during the early stage of implantation. The greater engraftment rate is particularly important because the success of cell-based vascular regeneration largely depends upon the number of cells that survive and engraft within the heart^42^.

Furthermore, SDF-eMSC-PAs not only promote vascular regeneration but also contribute to vascular enlargement as well as the transition to functional blood vessels, which can be defined as an initial stage in blood vessel maturation. Histological analysis showed that the number of blood vessels with a diameter of > 5 μm, which is physiologically and functionally important, in both the infarct and the border zones, in the heart of the EC + PA group was substantially higher than that in other experimental groups, including the hiPSC-EC alone group. As additional evidence, the in vitro Matrigel plug assay results suggested that treatment with conditioned media derived from the SDF-eMSCs maintained the integrity of the hiPSC-EC-derived vessels for longer than 48 h compared with that of the untreated control vessels. More interestingly, the SDF-eMSC-PAs appeared to induce arterialization of blood vessels, possibly by stimulating the migration and recruitment of smooth muscle cells, as the number of vessels positive for both CD31 and α-SMA was significantly higher in the EC + PA group than in the other experimental groups. Collectively, these results clearly indicate that SDF-eMSC-PA substantially contributed to vascular stability and integrity.

In summary, we propose a novel strategy for vascular regeneration in ischemic hearts through hiPSC-ECs and SDF-eMSC-PA induction that not only promotes hiPSC-EC-based vasculogenesis but also stimulates angiogenesis of vessels in the host infarcted hearts. Hence, our novel therapeutic strategy based on a dual system can be used for vascular regeneration and heart repair.

## Supplementary information

Supplentary materials
