# Supplementary material for: Enhancement strategy for effective vascular regeneration following myocardial infarction through a dual stem cell approach
Source: Exp Mol Med. 2022 Aug 16;54(8):1165–78. doi: 10.1038/s12276-022-00827-8 (PMC9440102; doi:10.1038/s12276-022-00827-8)
Supplement: Supplementary file 1 — Supplentary materials [file 12276_2022_827_MOESM1_ESM.pdf]

Supplementary information

Enhancement strategy for effective vascular regeneration following myocardial infarction through a dual stem cell approach

Running title: A dual approach for effective vascular regeneration

Hyeok Kim[1,2,*], Soon-Jung Park[4,*], Jae-Hyun Park[1,3,*], Sunghun Lee[3], Bong-Woo Park[1,2], Soon Min Lee[5], Ji-Won Hwang[1,2], Jin-Ju Kim[1,2], Byeongmin Kang[6], Woo-Sup Sim[1,2], Hyo-Jin Kim[5], Seung Hwan Jeon[7], Dong-Bin Kim[8], Jinah Jang[6], Dong-Woo Cho[9], Sung-Hwan Moon[4,10,#], Hun-Jun Park[1,2,11,#], Kiwon Ban[3,#]

[1]Department of Biomedicine & Health Sciences, College of Medicine, The Catholic University of Korea, [2]Division of Cardiology, Department of Internal Medicine, Seoul St. Mary's Hospital, The Catholic University of Korea, [3]Department of Biomedical Sciences, City University of Hong Kong, [4]Research Institute, T&R Biofab Co. Ltd, [5]SL BIGEN Inc., [6]Department of Creative IT Engineering and School of Interdisciplinary Bioscience and Bioengineering, Pohang University of Science and Technology, [7]Department of Urology, College of Medicine, The Catholic University of Korea, [8]Division of Cardiology, Department of Internal Medicine, The Catholic University of Korea, [9]Department of Mechanical Engineering, Pohang University of Science and Technology, [10]Department of Animal Biotechnology, Sangji University, 83, Sangjidae-gil, Wonju-si 26339, Gangwon-do, Korea. [11]Cell Death Disease Research Center, College of Medicine, The Catholic University of Korea.

*** These authors contributed equally**

# Address for Correspondence:

Sung-Hwan Moon, PhD, Research Institute, T&R Biofab Co. Ltd, Siheung, Republic of Korea. E-mail: safe33msh@gmail.com; Hun-Jun Park, MD, PhD, Seoul St. Mary's Hospital, The Catholic University of Korea, 222 Banpo-daero, Seocho-gu, Seoul, 137-701, Republic of Korea. Email:

cardioman@catholic.ac.kr or Kiwon Ban, PhD, City University of Hong Kong, 83 Tat Chee Avenue, Kowloon, Hong Kong SAR. E-mail: kiwonban@cityu.edu.hk

Supplementary information

Supplementary materials and methods

Differentiation of induced pluripotent stem cell-derived endothelial cells

hiPSCs (CMC-hiPS-011) cell line was purchased from KNIH and maintained on Matrigel using the StemMACSTM iPSC-BREW (Milltenybiotec) medium. Subsequently, to initiate the differentiation into endothelial cells, hiPSCs were then seeded onto a basement Matrigel (Corning)-coated cell culture dish (Eppendorf) at 70,000 cells/cm2 dish. 5 μM Y-27632 (Tocris) was added for the first 24 h after passage. The medium was changed daily, and hiPSCs could grow in iPSC-BREW for 3 days until the cells were 60% confluent. On day 0, the cells seeded and treated with CHIR99021 (Tocris) 6 μM/ml and after day 1, the culture medium is replaced and the CHIR99021 2 μM/ml treated again in RPMI1640 + B27(-, insulin). From 2 to 5 days, Human-Endothelial-Serum-Free-Medium (Thermofiser) supplement cytokine (20 ng/ml bFGF (RnD systems), 10 ng/ml EGF (R&D systems), 10 μg/ml human plasma fibronectin (Thermofisher) with 50 ng/ml VGFG (R&D systems) replaced daily changed for 3days. After 5 days, the VEGF concentration in the HESFM culture was reduced to 30 ng/ml, and once every 2 days, the sorting was performed on the 7th day. To isolate CD 31 positive cells, sorting was performed with a sorted through a SH800S Cell Sorter flow cytometer with Cell sorter software Ver 2.1.5 (Sony Biotechnology). The $CD31^+$ cells were replated for culture on collagen-coated plates in HESF medium (Thermofisher).

Generation of hiPSC-ECs-GFP

hiPSC expressing GFP signal (hiPSC-GFP) was generation by using GFP (CAG, Puro) lentiviral particles (GenTarget Inc, LVP579). Subsequently, these were transfected to 4.0×10^5 of hiPSC in opti-MEM media (Life Technologies) supplemented with 8 μg/mL polybrene (Millipore) for 24 hours. hiPSC colonies expressing GFP signal were FACS sorted based on the expression of GFP at 3 days. The purified of hiPSC-GFP colonies were expanded for further differentiation into the Endothelial cells. Finally, hiPSC-GFP were differentiated into the hiPSC-ECs-GFP through the previously used ECs differentiation protocol.

Generation of SDF-1α expressing MSCs

BM-MSCs (Catholic MASTER Cells) were obtained from Catholic Institute of Cell Therapy (CIC, Seoul, Korea). In order to immortalize the MSCs, replication-incompetent lentiviral vectors containing hTERT and c-Myc were prepared and transduced into the cells. To produce immortalized MSCs expressing SDF-1α, replication-incompetent lentiviral vectors expressing the human SDF-1α were prepared and further transduced to immortalized MSCs. Then, SDF-eMSCs were isolated as a monoclonal cell population via the limiting dilution method. The final monoclonal cells were selected based on SDF-1α protein secretion, proliferation rate, and other MSC phenotypes. The SDF-eMSCs were cultured in low glucose-containing Dulbecco's modified Eagle's medium (DMEM, Gibco) supplemented with 10 % fetal bovine serum (FBS, Gibco), 10 ng/mL basic fibroblast growth factor (bFGF, Peprotech) and 2 μg/mL doxycycline (Clonetech) at 37°C and 5% CO_2.

FACS analysis

hiPSC-ECs were dissociated with 0.25% Trypsin–EDTA (Gibco) and then was washed with FACS solution (PBS supplement 2% FBS). The single cells were incubated with APC-conjugated mouse anti-human CD31 (R&D systems) and PE conjugated mouse anti-human CD144 (BD Biosciences) for 20 minutes at 4℃. Cells were washed three times in FACS solution. FACS analysis was performed using SONY® flow cytometer (SH800, Sony Biotechnology, Inc., Tokyo Japan) with sony software 2.1.5version.

Immunocytochemistry

The cells were fixed with 4% paraformaldehyde and permeabilized with 0.1% bovine serum albumin (Sigma) in PBS (Gibco) for 3 minutes. After treatment with 1% normal goat serum for 30 minutes, the cells were incubated with mouse anti-human CD31 (Merck), rabbit anti-human vWF (Abcam.) antibody for 12 hours at 4℃. The cells were washed with PBS and incubated with goat anti-rabbit Alexa 488 and goat anti-mouse 594 secondary antibodies (Molecular Probes Inc., Eugene, OR) for 1 hour. The cells were counterstained with DAPI (Invitrogen) and mounted with a glycerol-based mounting solution (VECTOR). All images were analyzed using an Eclips-Ti2 fluorescence microscope (Nikon, Japan).

Ac-LDL Uptake assay

The ability of hiPSC-ECs to incorporate DiI-acetylated-low-density lipoprotein (DiI Ac-LDL, Molecular Probes) was confirmed by incubating the cells with 10 ng/ml DiI-Ac-LDL in complete HESF media for 4 hours at 37℃. The cells were three times washed and fixed with 4% paraformaldehyde for 10 minutes at room temperature. The cells were counterstained with DAPI (Invitrogen) after washing the cells three times with PBS then images were analyzed using an Eclips-Ti fluorescence microscope (Nikon, Japan).

Enzyme-linked immunosorbent assay

Enzyme-linked immunosorbent assay (ELISA) was performed to confirm the levels of SDF-1α in SDF-eMSCs, BM-MSC, and SDF-eMSC patches. Three different passages of SDF-eMSCs and BM-MSC were used for the ELISA assay. The SDF-eMSCs were seeded at a density of 5 x 10^6 cells in 100 mm dish (Corning) with 10 mL complete media. After 48 h at 37°C in a 5% CO_2 incubator, the medium was changed to serum-free DMEM. The conditioned media were harvested 72 h after changing the serum-free DMEM and centrifuged to remove cell debris. The SDF-eMSC concentrations were estimated using a Human SDF-1α ELISA kit (R&D Systems), according to the manufacturer's instructions. The absorbance of the samples at 450 nm was measured using a SpectraMAX 190 (Molecular Devices) microplate reader.

Cell proliferation analysis

To determine the proliferation rate of the SDF-eMSCs, 4 x 10^5 cells were seeded into a T75 flask. After 3-4 days, the cells were harvested, and the proliferation rate was measured using the trypan blue (Gibco) exclusion method with a single-use hemocytometer (INCYTO). The cumulative population doubling level (PDL) of the SDF-eMSCs was determined by the summation of population doubling (PD). The PDL was calculated using (logN-logN0)/log2, where N is the number of harvested cells and N0 the number of seeded cells.

Real time PCR

The hiPSC-ECs (1 x 10^5) were seeded on a 6-well plate containing different types of media (EGM, BM-MSC-CM, and SDF-eMSC-CM). The media were changed daily. After 72 hours, the total RNAs were

extracted by the addition of 0.5 mL of TRIzol reagent (Life Technologies) to cells on a plate as described in the manufacturer's instructions. One microgram of RNA was subjected to cDNA synthesis with SuperScriptTM Reverse Transcriptase IV and random primers (Invitrogen). SYBR® Green PCR Master Mix (Applied Biosystems) was used to detect the accumulation of PCR products during cycling with the ABI Real-time PCR StepOne Plus (Applied Biosystems). Real-time reverse transcription-polymerase chain reaction (RT-PCR) was carried out in triplicate in at least three independent experiments. Oligonucleotide primers were designed using real-time RT-PCR system sequence detection software v2.3 (Applied Biosystems) and their sequences are listed in Table 1. Fold differences in the expression level of each gene were calculated for each treatment group using CT values normalized to transcript levels of the housekeeping gene, 18S rRNA or GAPDH, according to the manufacturer's instructions.

Endothelial cell migration assay

The hiPSC-ECs and HUVEC cells (3.5×10^4) were seeded onto the upper layer of Transwell inserts (8 μm pore) with the EBM basal medium and then placed onto a 24-well plate containing the media (EGM, EBM, BM-MSC-CM, and SDF-eMSC-CM). The cells were then allowed to migrate for 7 h in 37°C with 5% CO_2. The migrated cells were fixed in 4% PFA for 10 minutes, followed by staining with 0.1% crystal violet (Sigma) for 10 minutes. After washing the Transwell membrane with distilled water, the upper side of the membrane was gently swiped with a cotton swab to remove non-migrated cells. The migrated cells were counted using a light microscope and the stained area of the membrane was measured using ImageJ software.

Tube formation assay

Geltrex™ LDEV-Free Reduced Growth Factor Basement Membrane Matrix (500 μl, Geltrex™ LDEV, Thermo Fisher) was added to the 24-well plate and solidified by incubation at 37°C for 30 minutes. Next, 1 x 10^5 cells of hiPSC-ECs and HUVECs were plated onto each Geltrex-coated well containing different types of media (EGM, EBM, BM-MSC-CM, and SDF-eMSC-CM) and then incubated at 37°C with 5% CO_2 for 9, 24, 48 h. After removing the media, 4% PFA was added for fixation. The cells were visualized using a light microscope and the formation of tube structures was calculated using ImageJ software.

Releasing kinetics of SDF-eMSC-PA

The long-term release kinetics of SDF-eMSC-PA were evaluated via human SDF-1α ELISA with conditioned media over time. The patches were made of hdECM (100 µl) with SDF-eMSCs (1 x 10^6) and human SDF-1α cytokines (50, 100, and 300 ng/mL) on a disk-shaped PCL (diameter: 1 cm). The patches were cultured in low glucose-containing Dulbecco's modified Eagle's medium (DMEM, Gibco) supplemented with 10 % fetal bovine serum (FBS, Gibco), 5 ng/mL basic fibroblast growth factor (bFGF, Peprotech) at 37°C and 5% CO_2. The media were changed every 2 days, and conditioned media of patches were obtained by accumulating for 72 h at 0, 7, 14, 21 and 28 days at each time point.

Neonatal cardiomyocyte isolation

Neonatal rat cardiomyocytes were isolated from 1-day-old Sprague-Dawley rat hearts, following the guidelines and under the approval of the Animal Care and Use Committee at the Catholic University of Korea. Ventricular tissue was incubated with 0.1% trypsin solution (Welgene) in Hanks' balanced salt solution (HBSS; Welgene) with gentle agitation at 4°C overnight. The next day, the digested tissue was transferred to a petri dish on ice, minced, and collected into a conical tube. A digestion solution [5 mL of collagenase B (1 mg/mL) in HBSS; Roche] was added to the heart tissue and triturated using a 5 mL pipette 15 times and incubated in a 37°C water bath for 5 min with gentle agitation. The supernatant containing the cardiomyocytes was transferred to isolation media [DMEM containing 20% FBS (Gibco) with 1X antibiotic-antimycotic (Gibco)], and the undigested heart tissue was resuspended in 10 mL of digestion solution. An additional digestion step was performed, and the cell-containing supernatant was collected. The digestion step was repeated five times, followed by centrifugation for 10 min at 1500 rpm and washing of the cell pellet in isolation media, followed by centrifugation for 5 min at 1500 rpm and resuspension of the cell pellet in DPBS for use in Percoll (GE healthcare, 17-5445-02) density-gradient centrifugation. Contaminating fibroblasts and ECs were removed by a Percoll density-gradient centrifugation 30 min at 3000 rcf 4°C. Next, the cardiomyocytes were transferred to a new conical tube, resuspended in isolation media, followed by centrifugation for 10 min at 1500 rpm and washing of the cell pellet in isolation media. Additional centrifugation for 5 min at 1500 rpm and resuspension of the cell pellet in isolation media were performed.

The cells were plated on 0.1 % gelatin (Welgene)–coated culture plates at a density of 1 × 10^5 cells/cm^2, and cultured at 37°C and 5% CO_2. FBS was excluded to suppress the growth of non-cardiomyocytes, and the medium contained 5% horse serum (Sigma, H1138) and 1X antibiotic-antimycotic (Gibco). The culture medium was changed every other day.

ROS protection assay

ROS protection assay of hiPSC-ECs, and HUVECs and NRCMs was conducted using a Live/Dead Viability kit (Invitrogen, L3224) and CCK-8 reagent (Dojindo Laboratories). The H2O2 (Sigma, 216763) reagents were dilutions of culture media, which included different types (EBM with 5% FBS or low-glucose DMEM with 5% horse serum, 30% of BM-MSCs, and SDF-eMSCs conditioned media). The total H2O2 concentration was 500 μM (NRCMs and HUVECs) and 700 μM (hiPSC-ECs). After treatment with H2O2 solution at 37°C and 5% CO_2 for 2 h, the media were changed to phenol red-free EBM or DMEM with a calcein 2 M and ethidium homodimer-1 dye 4 M mixture for the Live/Dead assay. Culture was performed at room temperature for 20 min. The cells were imaged using a fluorescence microscope (Nikon). Images were acquired under identical acquisition settings and processed using ImageJ. The viability of cells was assessed using the CCK-8 assay. The media were changed to phenol red-free EBM or DMEM. The CCK-8 reagent was added to 10% of the total media volume with 5% FBS and then incubated at 37°C with 5% CO_2 for 2 h. After 2 h incubation at 37°C, the absorbance at 450 nm was measured using an Elisa reader (Spectramax Plus384, MSD).

Immunohistochemistry

At the time of sacrifice, hearts were perfused with IB4 conjugated-rhodamine from Griffonia simplicifolia for 15 min at room temperature. The hearts were then fixed in 4% paraformaldehyde overnight and then blocks were made. The heart was cross-sectioned into 5 μm sections starting at the top of the apex using a microtome (Leica, RM2255, Germany). The sections were stored at -20°C before use. After deparaffinization and rehydration, antigen retrieval with target retrieval solution (DAKO) was performed in a humid chamber. The sections were blocked and incubated with diluted primary antibody (Dako) at 4°C overnight. The primary antibody used in this study was mouse anti-cTnT (Abcam; 1:200), rabbit anti-CD31

(Abcam; 1:200) and mouse anti-α-SMA (Sigma-Aldrich; 1:200). After washing three times with 1% Tween 20 in PBS, the samples were incubated with secondary antibody for 90 min at room temperature in the dark. The secondary antibody used in this study was anti-mouse Alexa Fluor 488 (Invitrogen; 1:500), anti-rabbit Alexa Fluor 594 (Invitrogen; 1:500) and anti-mouse Alexa Fluor 647 (Invitrogen; 1:500). After washing three times with PBS, the sections were stained with DAPI solution (VectaShield) for nuclear staining and then mounted on slides. The number of capillaries were counted in five random microscopic fields using a fluorescence microscope (Nikon) and expressed as the number of capillaries per square millimeter of tissue area.

Supplementary figures

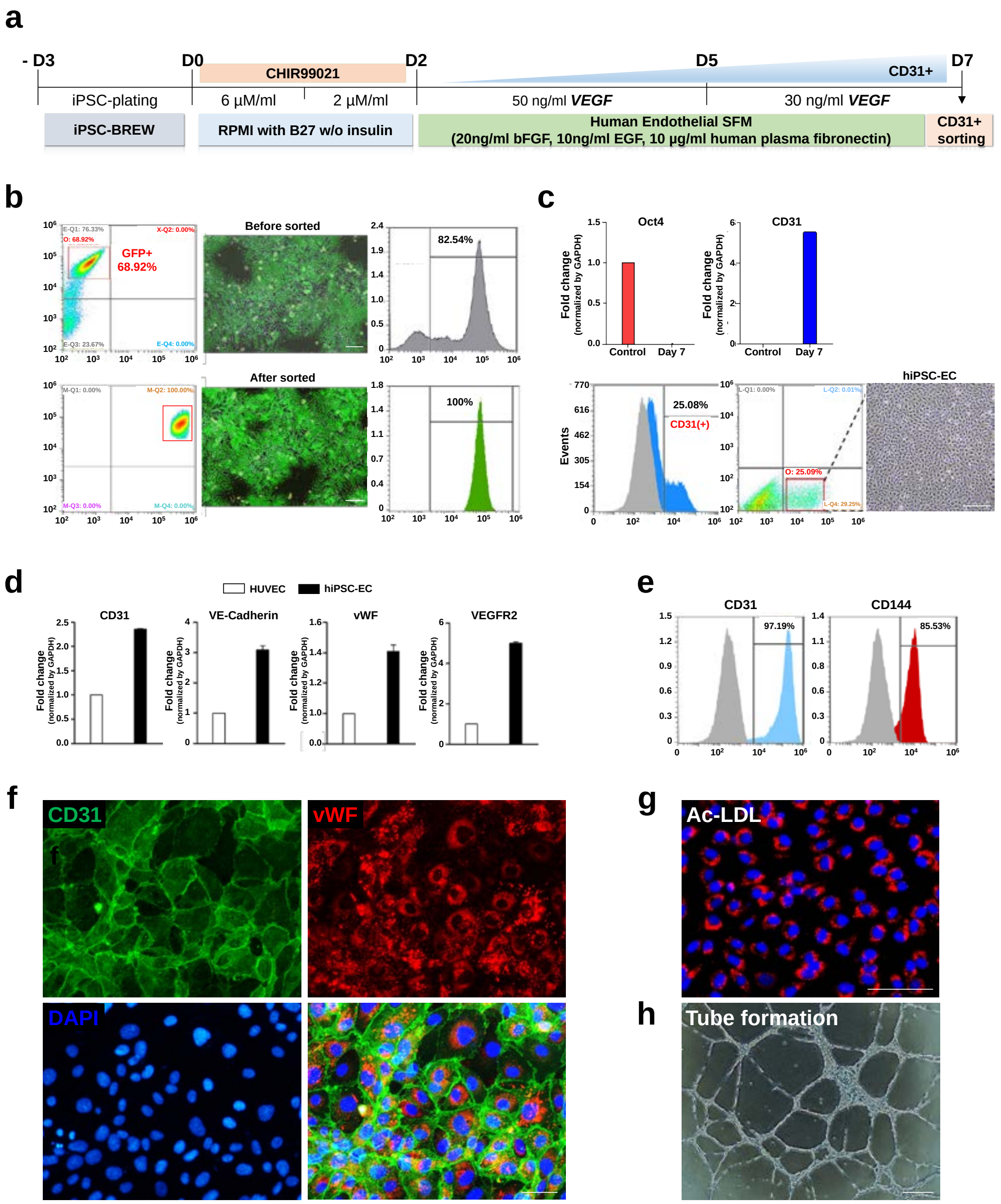

Supplementary Fig 1. Differentiation of ECs from human induced pluripotent stem cells (hiPSCs). a Schematic representation showing the protocol for Endothelial cells (EC) differentiation from hiPSCs. **b** Representative fluorescence-activated cell sorting (FACS) plots showing the percentage of GFP positive hiPSCs (hiPSC-GFP). The GFP positive hiPSCs (hiPSC-GFP) were sorted out for subsequent expansion and further differentiation into the ECs. Scale bar: 100 µm. **c** qRT-PCR analyses results of differentiating hiPSCs at differentiation day 7. Data were normalized by GAPDH. n=3. Sorted hiPSC-EC lineage cells using CD31 antibody $CD31^+$-hiPSC-ECs and their cellular morphology. Scale bar: 500 µm. **d** qRT-PCR analyses results of $CD31^+$-hiPSC-ECs. Data were normalized by GAPDH and expressed relatively to HUVECs. n = 3. **e** Flow cytometry analysis results of $CD31^+$-hiPSC-ECs. **f** Immunostaining results of $CD31^+$-hiPSC-ECs. CD31 (green, FITC), Von Willebrand Factor (red, TRITC), and nuclei stained with DAPI (blue). Scale bar: 100 µm. **g-h** The results of ac-LDL uptake analysis and tube formation assay using $CD31^+$-hiPSC-ECs. Scale bar: (**g**) 200 µm and (**h**) 100 µm.

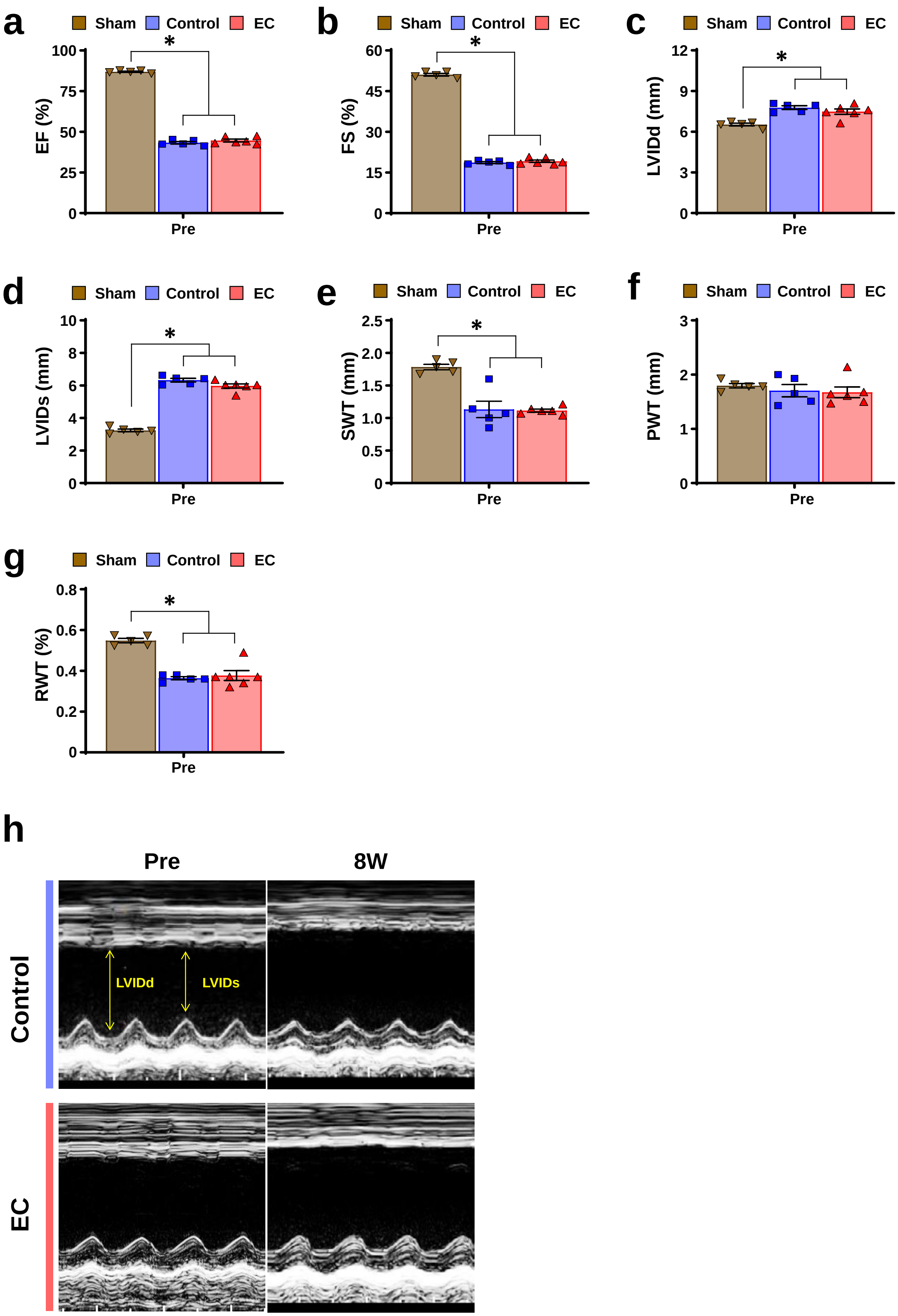

Supplementary Fig 2. Verification of MI modeling and representative image of M-mode. a Left ventricular ejection fraction (EF). **b** Left fractional shortening (FS). **c** Left ventricular internal diastolic dimension (LVIDd). **d** Left ventricular internal systolic dimension (LVIDs). **e** Septal wall thickness (SWT). **f** Posterior wall thickness (PWT). **g** Relative wall thickness (RWT). **h** Representative images M-mode at pre (1-week post-MI but prior to intervention) and at 8 week after hiPSC-ECs injection, n= 5-6. *p<0.05.

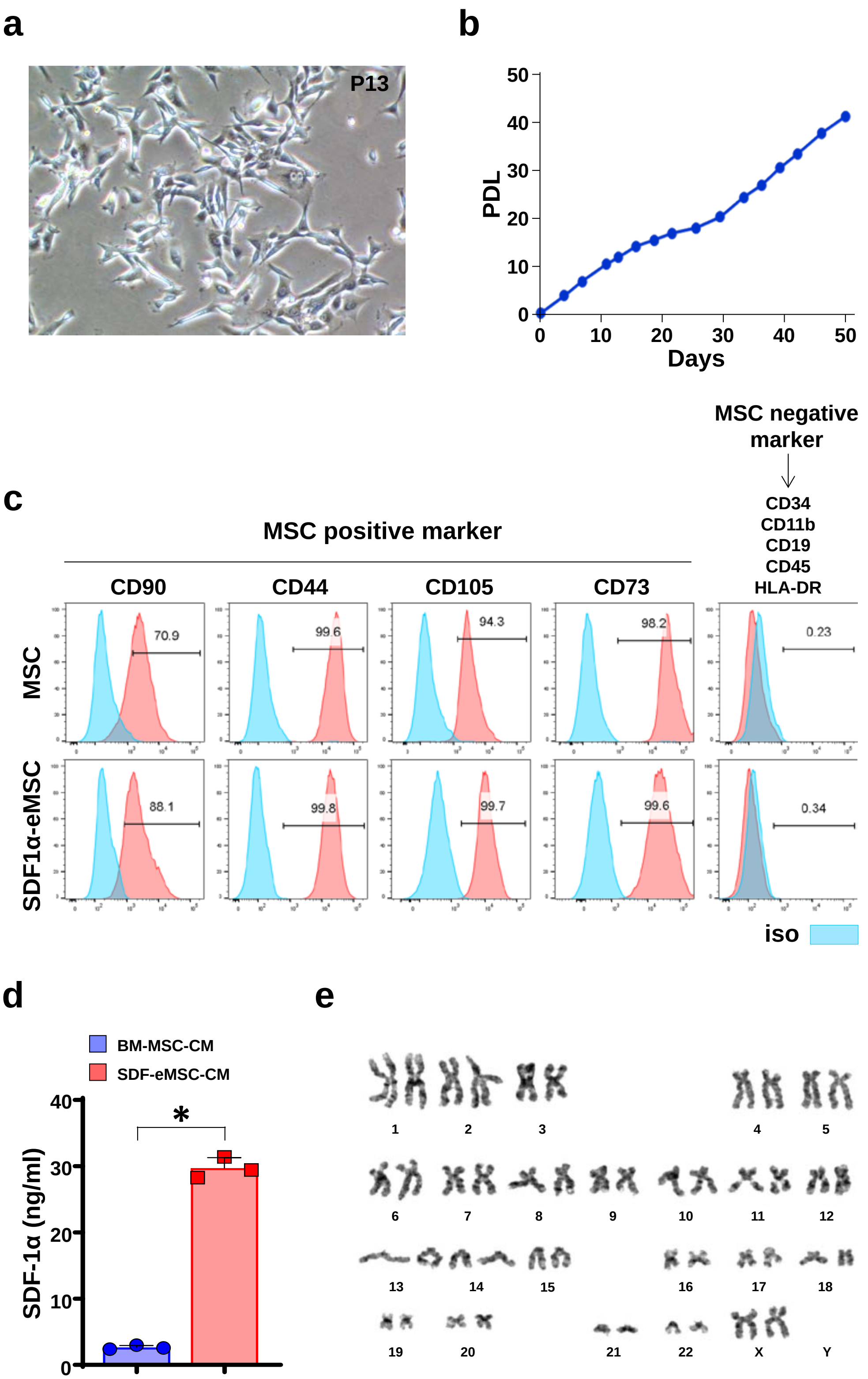

Supplementary Fig 3. Cellular characterization of SDF-eMSCs. a Morphology of SDF-eMSCs. **b** Cell proliferation rate of SDF-eMSCs. n=3. **c** Flow cytometry analyses show that SDF-eMSC express specific markers for MSCs such as CD90, CD44, CD105 and CD73. n=3. **d** SDF-1α secretion of SDF-eMSCs compared with normal BM-MSCs measured by human SDF-1α ELISA kit. n=3. **e** The results of karyotyping with SDF-eMSCs. SDF-eMSC: Engineered stromal derived factor-1α expressing MSCs.

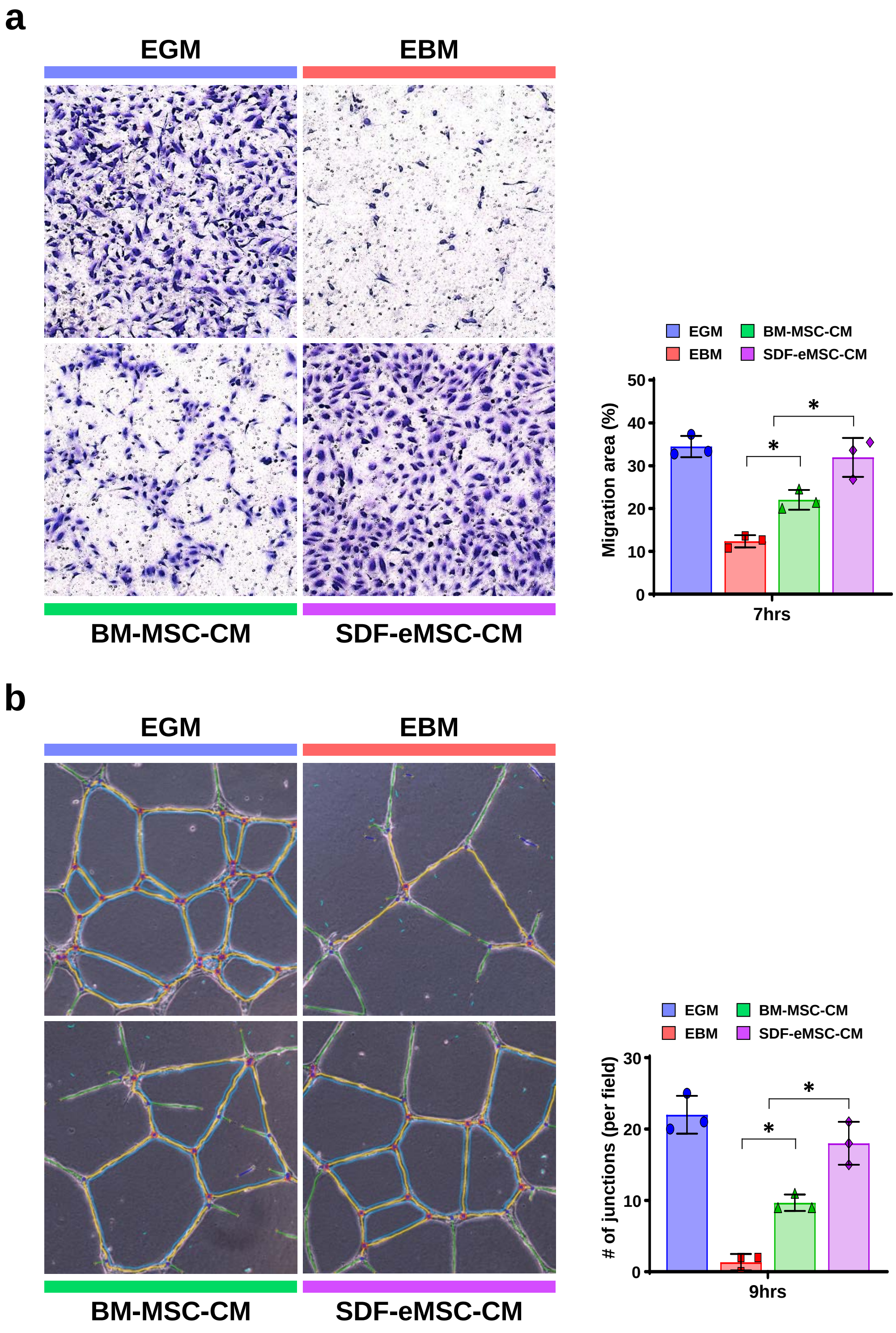

Supplementary Fig 4. SDF-eMSCs enhance angiogenic potential of HUVECs in vitro. a EC migration assay. Representative images of migrated HUVECs, and quantification of migrated area (%). The HUVECs were placed in transwell (Top), and regular media (EGM, EBM) or the conditioned media (CM) collected from different cell sources (BM-MSC-CM and SDF-eMSC-CM) were placed in transwell (Bottom) for 7 hours. n=3. *p<0.05. **b** Tube formation assay. The HUVECs were cultured in 24-well plates coated with Geltrex™ with regular media (EGM, EBM) or the conditioned media (CM) (BM-MSC-CM and SDF-eMSC-CM) for 9 hours. Representative images of tube formation and quantification summary for number of junctions. n=3. *p<0.05.

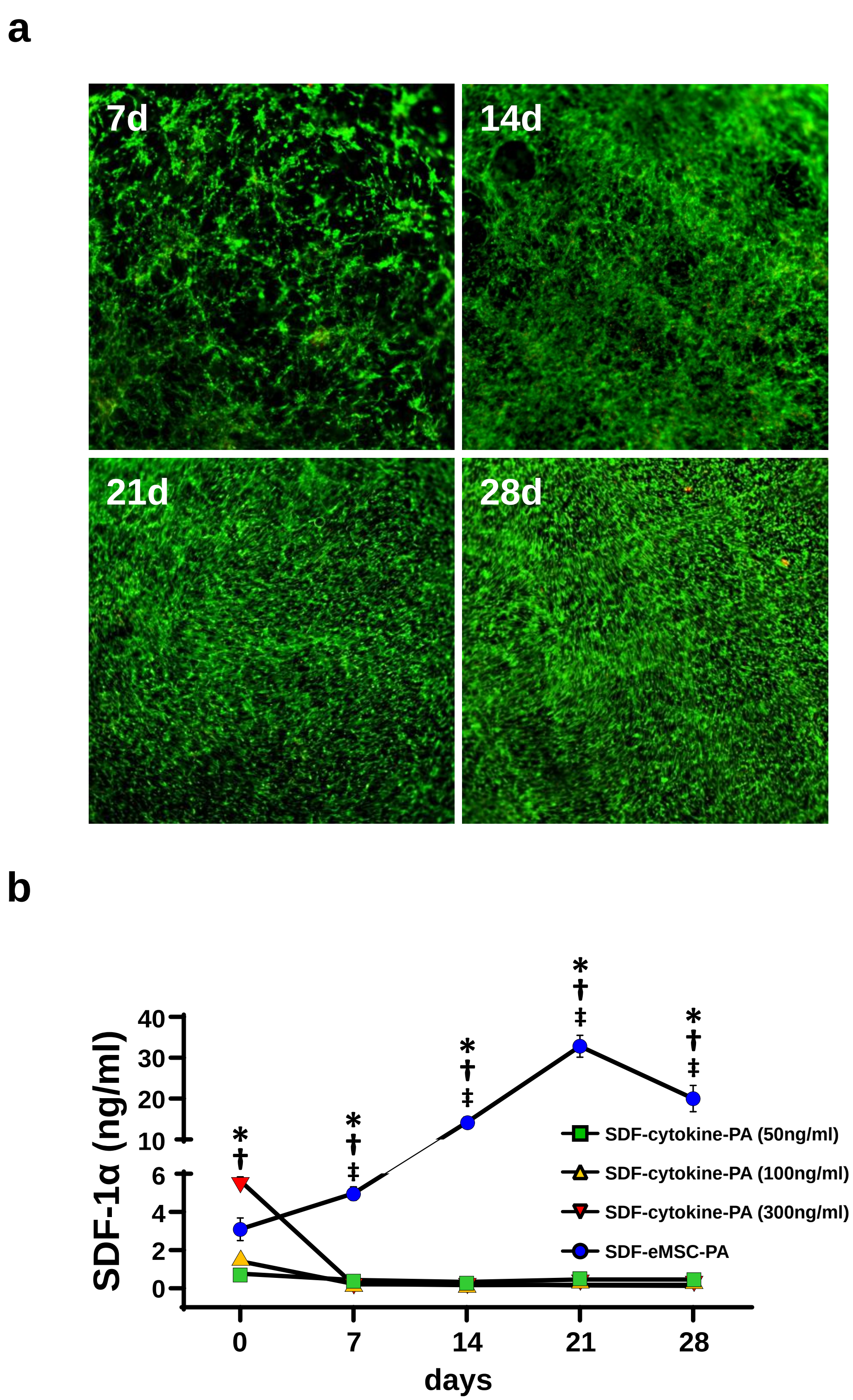

Supplementary Fig 5. Releasing kinetics of SDF-eMSC-PA. a Live & Dead staining of SDF-eMSCs within the hdECM patch over time. **b** SDF-1α cytokine expression of SDF-eMSC-PA or SDF-1α cytokine-PA. SDF-1α cytokine released from the patch was accumulated for 3 days at each time point and measured by ELISA. n=3 *p<0.05 compared with SDF-cytokine-PA group (50ng/ml); †p<0.05 compared to SDF-cytokine-PA (100ng/ml); ‡p<0.05 compared to SDF-cytokine-PA group (300ng/ml). SDF-eMSC-PA: Patch encapsulating SDF-eMSC. SDF-cytokine-PA: Patch encapsulating SDF-cytokine-PA (50ng/ml) or (100ng/ml).

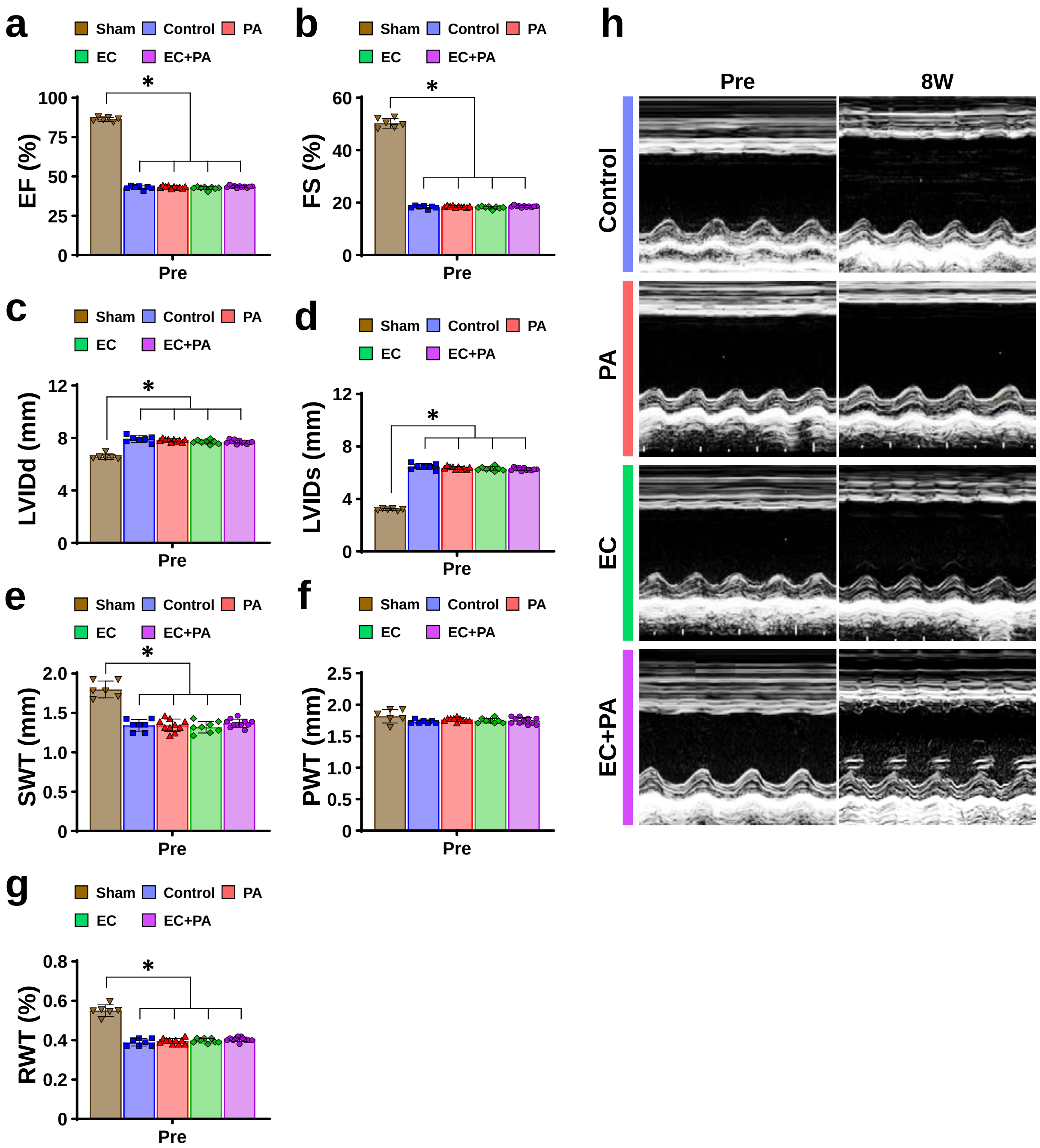

Supplementary Fig 6. Verification of MI modeling and representative image of M-mode.

a Left ventricular ejection fraction (EF). **b** Left fractional shortening (FS). **c** Left ventricular internal diastolic dimension (LVIDd). **d** Left ventricular internal systolic dimension (LVIDs). **e** Septal wall thickness (SWT). **f** Posterior wall thickness (PWT). **g** Relative wall thickness (RWT). **h** Representative images M-mode of four experimental groups at pre (one-week post-MI prior to intervention) and at 8 weeks post cell treatment. n= 7-11. *p<0.05.

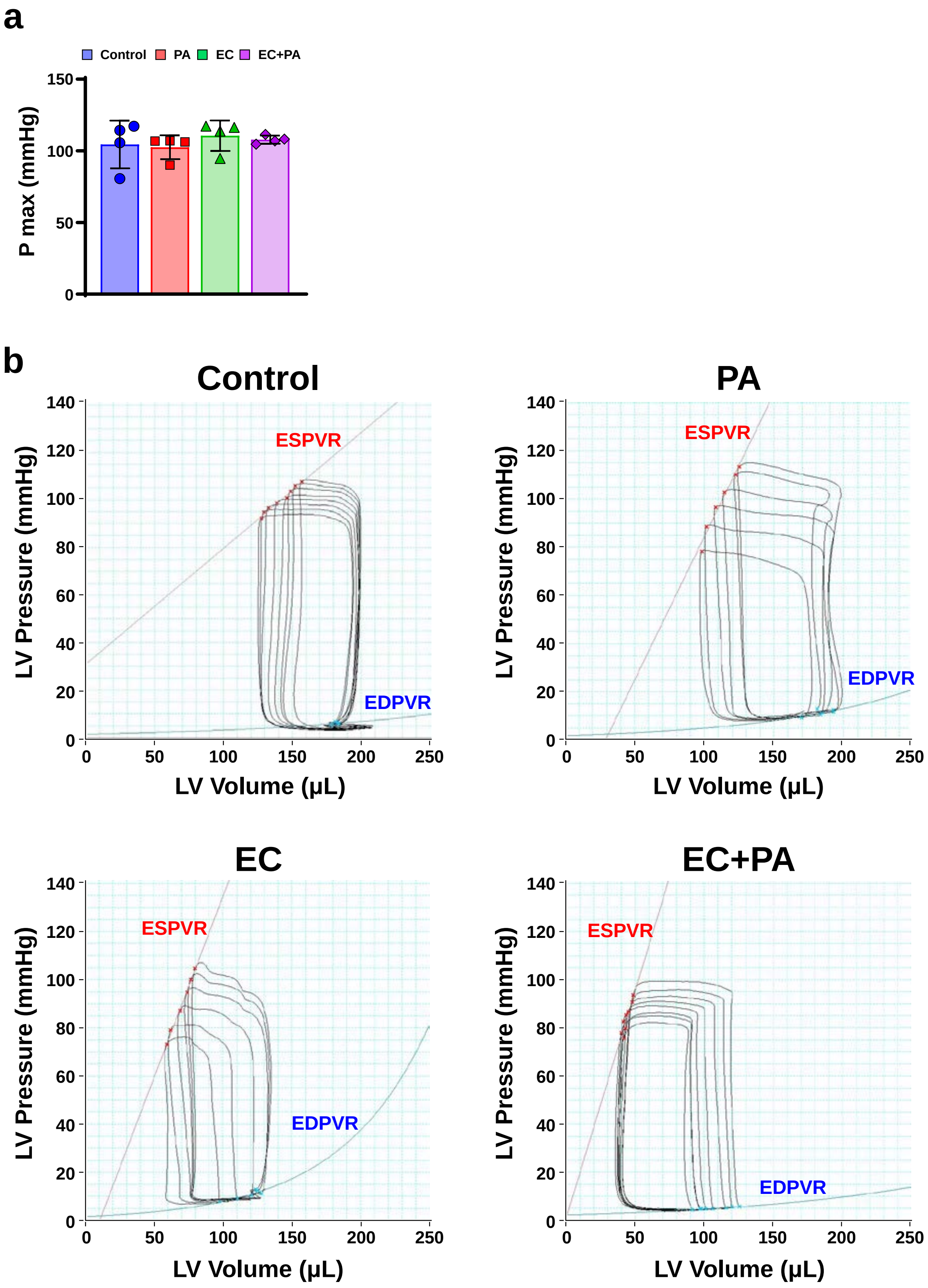

Supplementary Fig 7. Hemodynamic instinct cardiac function.

a Pressure max (P max) at the end-systole. n=4. *p<0.05. Representative image of intrinsic cardiac function in the: **b** control group, SDF-eMSC-PA only, hiPSC-ECs only, and hiPSC-ECs + SDF-eMSC-PA. n=4.

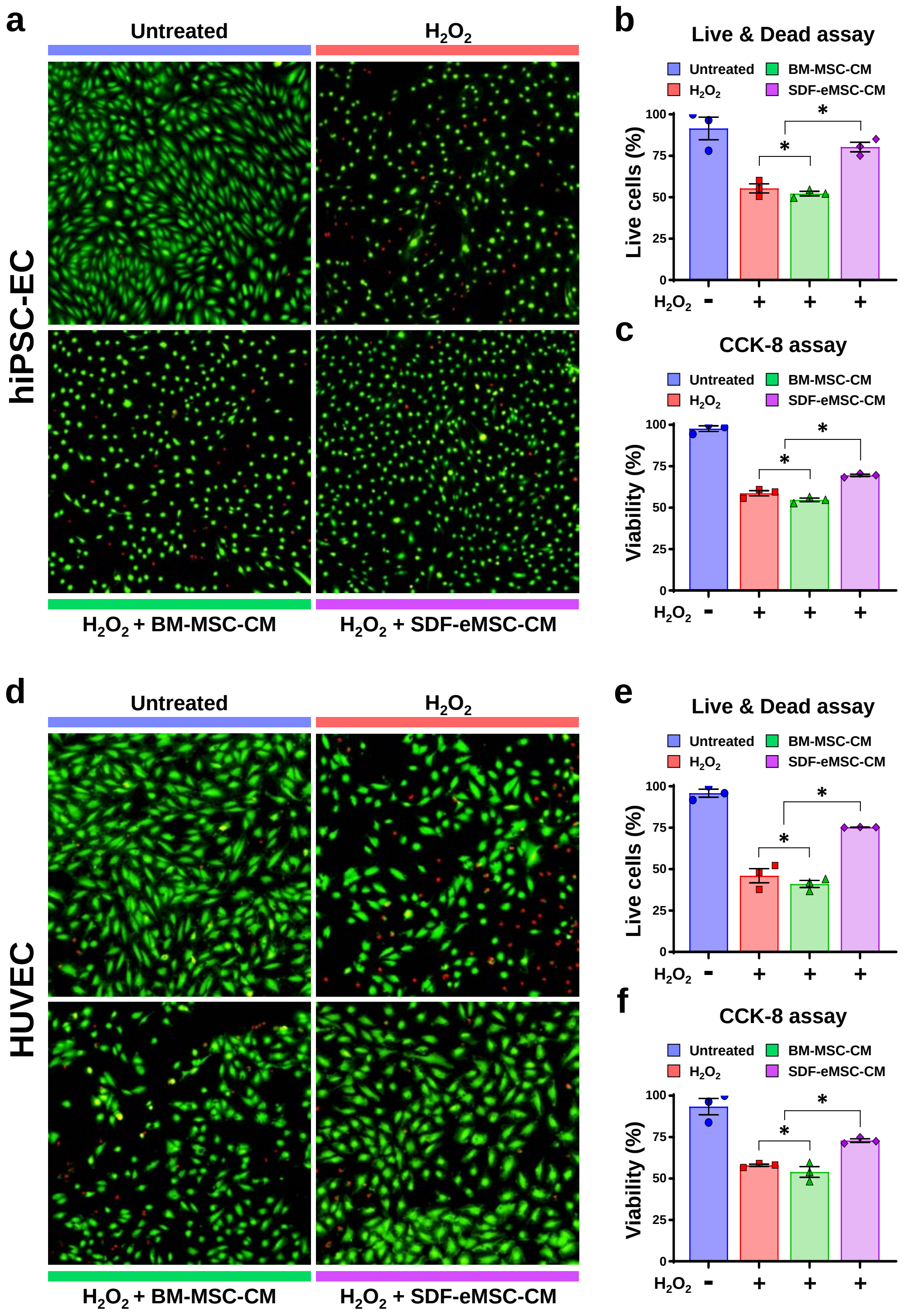

Supplementary Fig 8. Direct cytoprotective effects of the SDF-eMSC-conditioned medium on endothelial cells undergoing simulated ischemic injury. Treatment with conditioned media from BM-MSCs (BM-MSCs-CM) or SDF-eMSCs (SDF-eMSC-CM) increased survival of hiPSC-ECs (**a-c**) or HUVECs (**d-f**) after H2O2 (500 μM) treatment for 2 hours as determined by the Live & Dead assay and CCK-8 assay. **a** Representative image of Live & Dead assay with the hiPSC-ECs. **b** Quantification summary of the Live & Dead assay. **c** Quantification summary of the CCK-8 assay. **d** Representative image of Live & Dead images with the HUVECs. **e** Quantification summary of the Live & Dead assay. **f** Quantification summary of the CCK-8 assay. n=3 for all tests. *p<0.05.

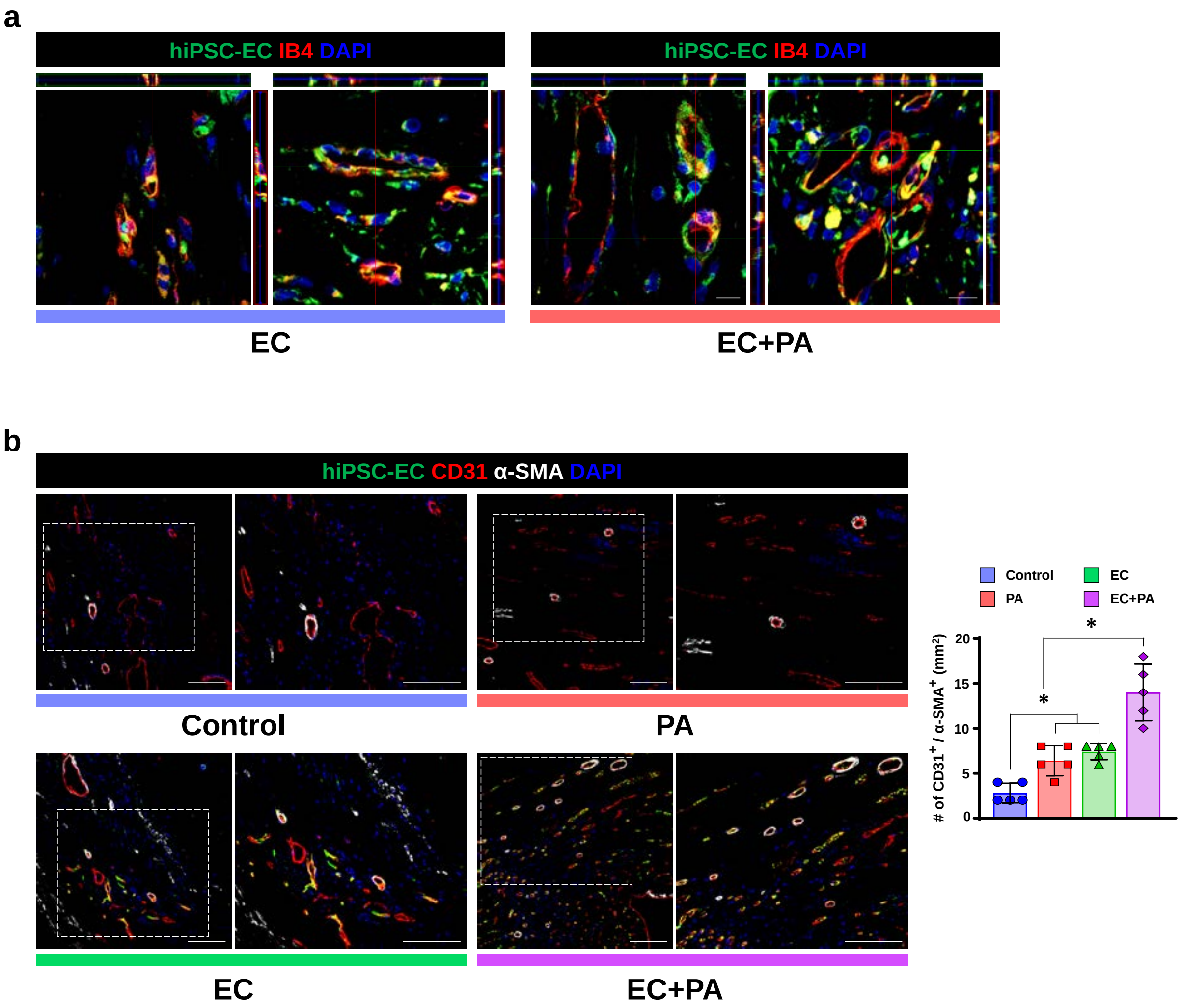

Supplementary Fig 9. Representative images of de-novo vessels formed by hiPSC-ECs. a Immunostaining images with hiPSC-ECs-GFP (green), IB4-rhodamine (red) and DAPI (blue). Scale bars: 10 μm. **b** Representative image of arterioles formed by hiPSC-ECs-GFP (green), CD31 (red), α-SMA (white) and DAPI (blue) and their quantification summary. n=5. *$p<0.05$. Scale bars: 50 μm.

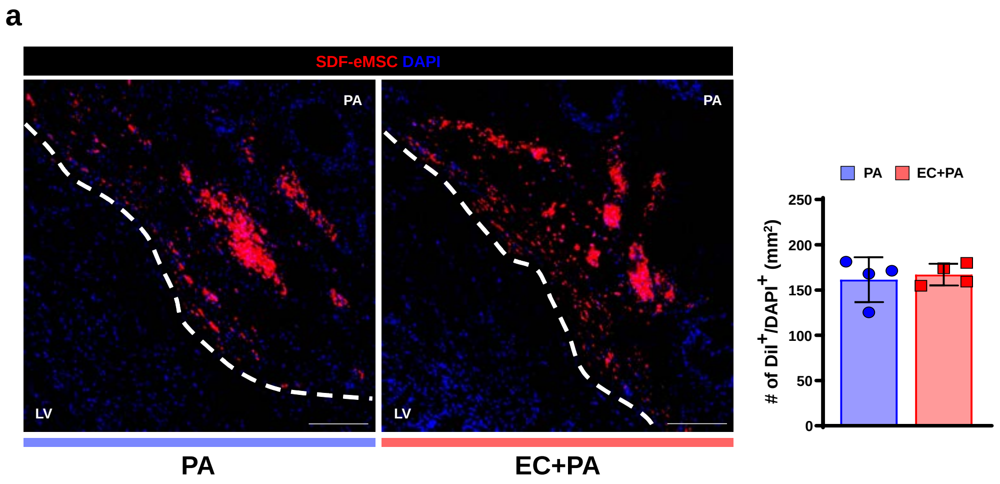

Supplementary Fig 10. Cellular behavior of SDF-eMSCs within 3D hdECM patch. a Representative image of retention of SDF-eMSCs labeled DiI (1,1'-Dioctadecyl-3,3,3',3'-Tetramethylindocarbocyanine Perchlorate) within 3D hdECM patch at 8 weeks post-cell treatment and quantification of the number of DiI positive. n=4. Scale bars: 200 µm.

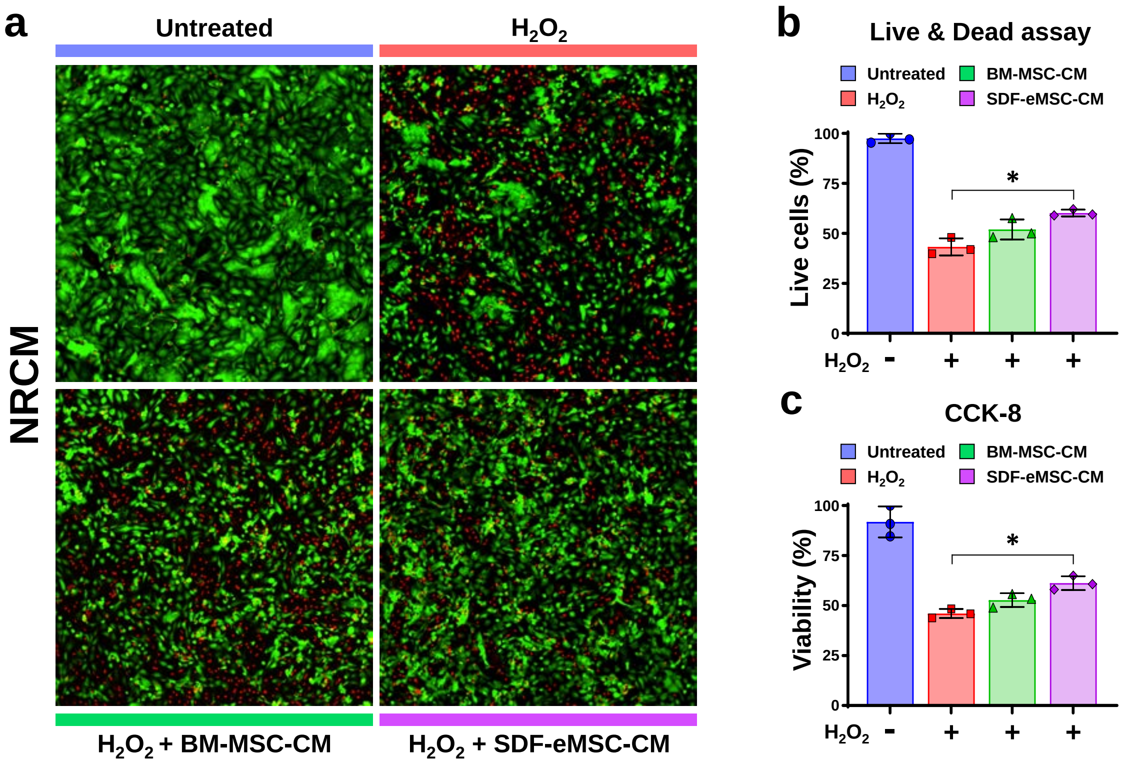

Supplementary Fig 11. Direct cytoprotective effects of the SDF-eMSC-conditioned medium on cardiomyocytes undergoing simulated ischemic injury. Treatment with conditioned media from BM-MSCs (BM-MSCs-CM) or SDF-eMSCs (SDF-eMSC-CM) increased survival of cultured neonatal rat cardiomyocyte (NRCMs) after H2O2 (500 µM) treatment for 2 hours as determined by the Live & Dead assay and CCK-8 assay. **a** Representative image of Live & Dead assay with the NRCMs. **b** Quantification summary of the Live & Dead assay. **c** Quantification summary of the CCK-8 assay. n=3 for all tests. *$p<0.05$.

Supplementary Table 1. Primer sequences used for qRT-PCR Analysis

Gene	Primers	
	**Forward**	**Reverse**
**GAPDH**	AGTGCCAGCCTCGTCTCATA	GTAACCAGGCGTCCGATACG
**SDF-1α**	AAGTGTGCATTGACCCGAAG	GTTTCAGAGCTGGGCTCCTAC
**VEGF**	ACGAAAGCGCAAGAAATCCC	CTCCAGGGCATTAGACAGCA
**HGF**	CGACAGTGTTTCCCTTCTCG	ATTGAGAACCTGTTTGCGTTTCT
**TIE2**	CCAGGATGGCAGGGGCTCCA	GGTAGCGGCCAGCCAGAAGC
**vWF**	CCTTGGTCACATCTTCACATTCAC	TCATTGGCTCCGTTCTCATCAC
**eNOS**	AGATGGTCAACTATTTCCTGTC	GCCACTTCTTTAAAGGTCTTC
**KDR**	GTTCTTCTGGCTACTTCTTGTCATC	GCATCATAAGGCAGTCGTTCAC
**CD31 (PECAM)**	GCAGTGGTTATCATCGGAGTG	CGTTGTTGGAGTTCAGAAGTGG
**VE-Cad**	TGGTGCCTATCTGCCTGGAG	CTTGGAGTGGAGTATGGAGTTGG
**E-SELE**	CGAAGGGTTTGGTGAGGTGT	AGCTGAAGGCACAAGAGGAC
**ICAM-1**	CAGCTTCTCCTGCTCTGCAA	CAATCCCTCTCGTCCAGTCG
